# Host matrix metalloproteinases in cerebral malaria: new kids on the block against blood–brain barrier integrity?

**DOI:** 10.1186/2045-8118-11-1

**Published:** 2014-01-27

**Authors:** Manuela Polimeni, Mauro Prato

**Affiliations:** 1 Dipartimento di Oncologia, Università di Torino, Torino, Italy; 2 Dipartimento di Neuroscienze, Università di Torino, C.so Raffaello 30, 10125 Torino, Italy; 3 Dipartimento di Scienze della Sanità Pubblica e Pediatriche, Università di Torino, Torino, Italy

**Keywords:** Cerebral malaria (CM), *Plasmodium*, Blood–brain barrier (BBB), Matrix metalloproteinases (MMPs), Inflammation

## Abstract

Cerebral malaria (CM) is a life-threatening complication of *falciparum* malaria, associated with high mortality rates, as well as neurological impairment in surviving patients. Despite disease severity, the etiology of CM remains elusive. Interestingly, although the *Plasmodium* parasite is sequestered in cerebral microvessels, it does not enter the brain parenchyma: so how does *Plasmodium* induce neuronal dysfunction? Several independent research groups have suggested a mechanism in which increased blood–brain barrier (BBB) permeability might allow toxic molecules from the parasite or the host to enter the brain. However, the reported severity of BBB damage in CM is variable depending on the model system, ranging from mild impairment to full BBB breakdown. Moreover, the factors responsible for increased BBB permeability are still unknown. Here we review the prevailing theories on CM pathophysiology and discuss new evidence from animal and human CM models implicating BBB damage. Finally, we will review the newly-described role of matrix metalloproteinases (MMPs) and BBB integrity. MMPs comprise a family of proteolytic enzymes involved in modulating inflammatory response, disrupting tight junctions, and degrading sub-endothelial basal lamina. As such, MMPs represent potential innovative drug targets for CM.

## Introduction

Human malaria is a widespread infectious disease caused by *Plasmodium* protozoan parasites and is associated with high morbidity and mortality rates, resulting in 627,000 deaths among 207 million cases estimated in 2012 [1]. Human malaria is caused by five different *Plasmodium* species: *P. falciparum*, *P. malariae*, *P. ovale*, *P. vivax* and *P. knowlesi. P. falciparum* and *P. vivax* are the most common, correlating with the most severe forms of malaria and the highest death rate, whereas other *Plasmodium* species generally cause milder forms of malaria which are rarely fatal [1]. The majority of deaths occur among children under the age of five years living in sub-Saharan Africa, and in Southern/South-Eastern Asia and Central/Southern America where mortality mainly affects adults. Additionally, occasional cases are observed in non-immune adult travelers from developed countries returning from these areas. Despite the intense efforts made by the research community and the Global Eradication program [2], no effective vaccines or adjuvant therapies are available for complicated malaria. It is projected that in the next few years the dramatic issue of drug-resistant malaria could become a serious threat [3-5].

*P. falciparum* is unique in that it causes mature infected red blood cells (iRBCs) to sequester and adhere to microvascular beds in numerous organs. A paradigmatic complication of *falciparum* malaria is cerebral malaria (CM), which develops after iRBCs sequester in the microvasculature of the central nervous system (CNS). Unlike the other human malarial parasites which rarely cause neurological dysfunction, *P. falciparum*-induced CM often leads to death or severe neurological sequelae [6]. Curiously, *P. falciparum* appears to remain in the vascular space without ever entering the brain parenchyma, in contrast to other encephalitis-causing pathogens, such as *Trypanosoma* spp. or *Toxoplasma gondii*[7], thus raising question of how intravascular *Plasmodium* parasites are capable of inducing such a devastating neural dysfunction in CM.

Recent evidence suggests that a compromised integrity of the blood–brain barrier (BBB) results in a subsequent increase in BBB permeability which enables toxic soluble factors released either by host or parasite to cross this barrier and exert neurological effects. This review focuses on CM pathophysiology and novel insights from animal and human models into the role of BBB functional impairment in CM. Finally, we discuss the emerging role of host matrix metalloproteinases (MMPs), a family of proteolytic enzymes related to inflammation and BBB damage in CM, opening the possibility for discovery of new effective adjuvant therapies for CM.

### Pathophysiology of cerebral malaria

CM appears as a diffuse encephalopathy commonly presenting with headache, agitation, frank psychosis, seizures and impaired consciousness, and occasionally with brainstem signs or focal neurological signs such as hemiplegia and cranial nerve palsies [8,9]. According to the World Health Organization (WHO) clinical criteria, CM is defined as a potentially reversible, diffuse encephalopathy causing a Glasgow coma score of 11/15 or less, often associated with fitting, in the absence of other factors that could cause unconsciousness such as coexistent hypoglycemia or other CNS infections [10]. It is difficult to confirm diagnoses of CM in endemic areas because of overlapping infections such as bacterial meningitis in patients showing incidental malarial parasitaemia [11]. Children from areas endemic for malaria or non-immune adults traveling from developed countries are at higher risk for developing CM. On the contrary, CM is rarely encountered in > 10-year-old patients who have been exposed to *P. falciparum* since birth. Mortality ranges from 15–30%, and 11% of children display neurological deficits upon discharge [12].

**Figure 1:**
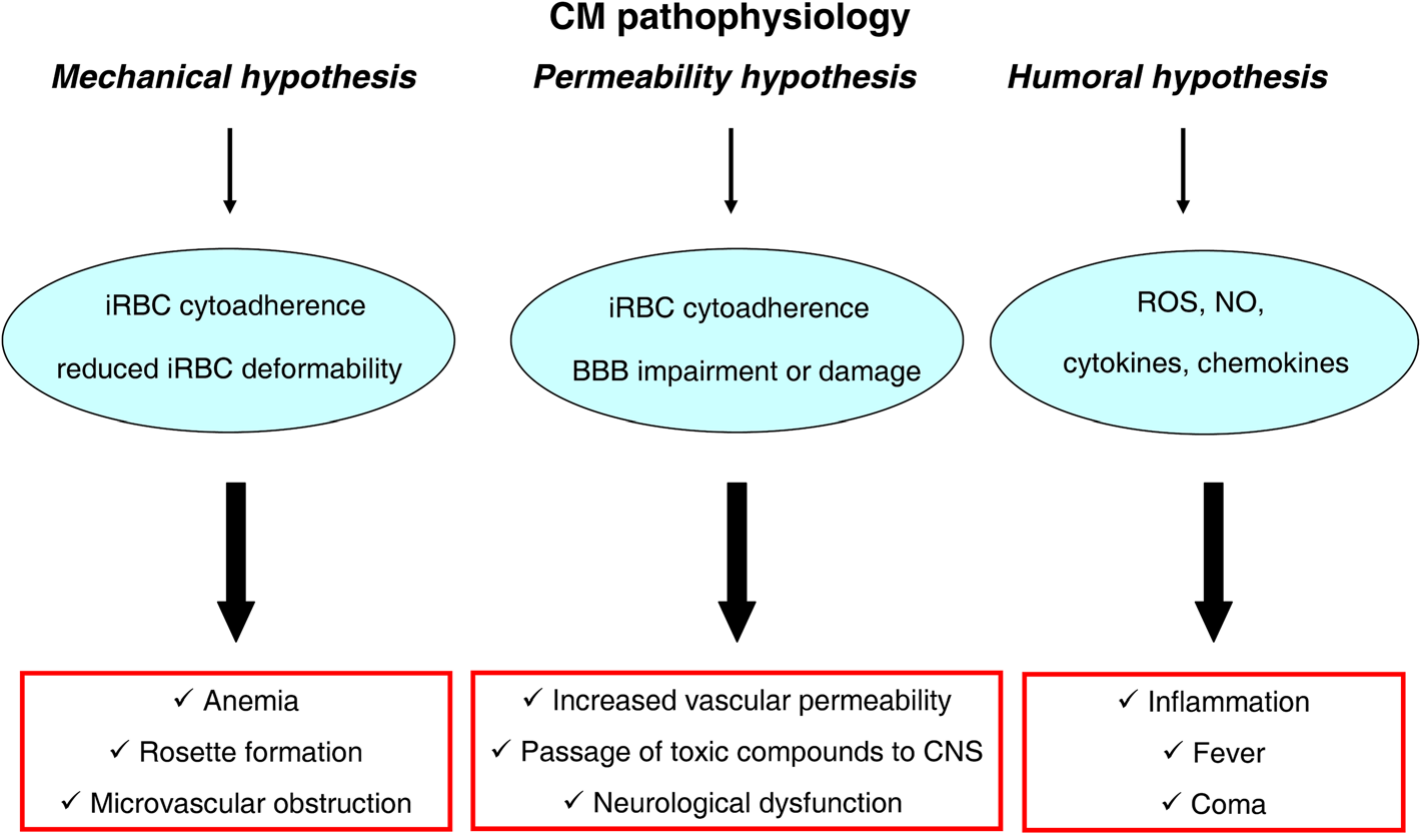
**Most commonly accepted hypotheses for pathophysiological mechanisms underlying clinical progress towards cerebral malaria (CM).** The diagram summarizes the three distinct hypotheses on CM etiology and their typical features: i) the mechanical hypothesis is associated with iRBC cytoadherence and their reduced deformability, causing following anemia, rosette formation and microvascular obstruction; ii) the permeability hypothesis is based on BBB impairment and subsequent increase in vascular permeability, allowing toxic compounds to reach the brain parenchyma and causing neurological dysfunction; iii) the humoral hypothesis focuses on the enhanced production by the host of pro-inflammatory molecules, including cytokines and chemokines, and other soluble factors such as ROS, which are putatively responsible for inflammation, fever and coma during CM.

The pathophysiological mechanisms underlying CM are not fully understood so far. As seen in Figure 1 and discussed in the next paragraphs, there are currently three distinct theories on the etiology of CM typical features: i) the mechanical hypothesis; ii) the permeability hypothesis; and iii) the humoral hypothesis [4,9,13-16]. It is possible that these theories are all pieces of that puzzle that need to be combined as they likely constitute more complementary than alternative models [6,17].

#### Mechanical hypothesis

The mechanical hypothesis proposes CM is caused by a mechanical obstruction of the cerebral microvasculature, with coma resulting from impaired brain perfusion [9,14,18]. Such a hypothesis was made after one of the first pathological studies on human CM showed that brain capillaries were packed with iRBCs [18]. In the mechanical hypothesis, specific interactions between iRBCs and vascular endothelium are thought to mediate sequestration of iRBCs within the brain resulting in removal from peripheral circulation [19-21]. The molecules involved in these interactions are parasite proteins expressed on iRBC surface, such as *P. falciparum* erythrocyte membrane protein-1 (PfEMP-1), and specific host receptors in the microvascular endothelium, including intracellular adhesion molecule-1 (ICAM-1), vascular cellular adhesion molecule-1 (VCAM-1), thrombospondin, CD36, and E-elastin [22-25].

Cytoadherence and decreased pliability are the main mechanisms underlying vascular obstruction [9,17-21]. It is speculated that cytoadherence evolved as a mechanism for the parasite to evade triggering a host immune response and being cleared from the spleen. Cytoadherence is also beneficial for the parasite as to provide an optimal environment of low oxygen tension for parasite growth. Decreased deformability along with increased membrane stiffness and rigidity of iRBCs are due to changes in the cytoskeleton triggered by growing intracellular parasites. Cell deformability has been indicated as a predictor of anemia development [26], whereas cell rigidity correlates with a higher fatality rate [27]. Another phenomenon occurring along with iRBC sequestration is rosetting, characterized by iRBCs forming a flower-like cluster around a non-iRBC, making a tight rigid structure [28]. Rosetting is more frequent in patients with CM than in those with uncomplicated malaria. However, rosette formation has also been reported for other *Plasmodium* strains (*P. vivax* and *P. ovale*) which do not cause CM [29]. Since rosetting occurs in all manifestations of the disease, it is not associated with severity or clinical outcome of CM [30]. One question the mechanical hypothesis by itself does not explain is why most patients recovering from CM do not show any evidence of ischemic brain damage [12].

#### Permeability hypothesis

The permeability hypothesis proposes that BBB damage is the underlying mechanism of CM, such that a leaky BBB allows toxic compounds to enter the brain and cause neurological dysfunction [9,13-15,31]. Several animal CM models have confirmed that the BBB is disrupted and that cerebral edema is present in CM, although this is less evident in humans [15]. Nevertheless, iRBCs remain attached to endothelium, without entering the brain parenchyma [7,9]. Interestingly, Adams and colleagues have suggested that iRBC cytoadherence might activate secondary signaling events similar to those occurring in leukocytes [32]. These secondary signaling events are thought to cause functional alterations in the BBB, which could allow toxic compounds to pass into the CNS. These events might be reversible, therefore explaining why neurological manifestations are just transient in most cases and why a large number of recovering patients lack neurological sequelae [32].

Enwonwu and colleagues implicated histamine as one of these toxic molecules that enters the brain parenchyma after BBB impairment and contributes to the neurological manifestions of CM [33-37]. The authors observed altered neural histidine uptake in children with severe *falciparum* malaria providing an explanation for the enhanced cerebral production of histamine [33]. They also found increased plasma levels of histamine in severe malaria patients, further supporting their hypothesis [34]. Moreover, the involvement of histamine in CM has also recently been confirmed in a murine model [35-37]. In this study, histidine decarboxylase-deficient mice were unable to synthesize free histamine and did not develop CM after infection with *P. berghei* ANKA. These mice displayed preserved BBB integrity, were void of iRBC aggregation in the brain vessels, and did not sequester CD4+ and CD8+ T cells [36]. Further investigation of histamine receptors revealed histamine-1-receptor (H1R) and histamine-2-receptor (H2R) are associated with severe malaria development [37], whereas histamine-3-receptor (H3R) has a neuroprotective role [36].

#### Humoral hypothesis

The humoral hypothesis is a natural extension of the permeability hypothesis. This hypothesis suggests that host factors such as leukocyte-derived cytokines and chemokines can enter the brain parenchyma after increased BBB permeability, thus causing CM symptoms such as fever and coma [9,13,14,16,38-40].

Effector cells including T cells, NK cells, and monocytes, along with inflammatory responses mediated by cytokines such as tumor necrosis factor-α (TNF-α), limphotoxin-α (LT-α), and interferon-γ (IFN-γ), are proposed to contribute to the development of murine CM [41-48]. However, the extent of their involvement and molecular mechanisms in human CM is still topic of debate [48,49].

CD8+ T cells have been reported to initiate BBB tight junction disruption and promote CNS vascular permeability under neuroinflammatory conditions [50-52]. Consistently, CD8+ T cell sequestration in cerebral microvessels and subsequent brain infiltration have been demonstrated in murine CM [43,44], where *Plasmodium* antigens can be cross-presented during infection by dendritic cells (DCs) [53,54] and brain endothelial cells in association with MHC class I molecules [55]. Recent human studies support the idea that malaria antigens can be transferred to endothelial cells [56]. However, it is currently unknown whether *Plasmodium*-specific CD8+ T cells are involved in the pathogenesis of human CM [57]. Furthermore, lymphocyte infiltration into brain parenchyma remains to be investigated [49].

TNF-α relevance in CM is also unclear. TNF-α involvement in murine CM was first proposed in 1987 [58]. Since then there have been numerous studies investigating TNF-α levels in CM mice albeit the results are inconsistent. For example, some works confirmed the association of high TNF-α levels with murine CM [59-61], whereas others argued against such correlation, finding LT-α and IFN-γ as more suitable markers [44,47,62,63]. To reconcile such discrepancies, it has been proposed that low concentrations of TNF-α could enhance parasite killing, whereas higher concentrations might be associated with increased incidence of murine CM [46]. However, data on TNF-α also appear inconclusive in human CM studies. Indeed, clinical studies tend to exclude any association between CM and increased plasma, serum or CSF levels of TNF-α [64-67], although a few works have proposed a correlation in two different Asian populations [68,69]. Instead, in some of these studies, high CXCL10/IP-10 plasma levels and low angiogenic factors such as vascular endothelial growth factor (VEGF) and angiopoietin-1 (Ang-1) in children with CM, predicted subsequent mortality [65,66,68]. Moreover, a protective role for IL-12 has been proposed in human CM [70,71].

Among soluble factors involved in CM, a critical role for nitric oxide (NO) has also been suggested. It was hypothesized that NO levels correlate with disease severity, since the sequestration of iRBCs might contribute to CM pathogenesis by causing hypoxia, which is related to enhanced production of cytokine-induced NO, compensatory vasodilatation, and subsequent brain volume increase [39]. However, activation of inducible NO synthase (NOS) might also serve protective functions, since NOS inhibits the side effects of brain indoleamine 2,3-dioxygenase (IDO) and quinolinic acid accumulation [72], although IDO systemic distribution is independent of malaria disease severity [73]. In a study performed on Tanzanian children infected with malaria, the plasma levels of NOS-suppressing IL-10 increased with disease severity, suggesting that a reduced NO production may contribute to CM [74]. Moreover, a genetic single nucleotide polymorphism found in the NOS2 promoter region causes elevated NO production and was significantly associated with protection against CM in Tanzanian and Kenyan children [75]. In line with these observations, Anstey and colleagues demonstrated that decreased NO production was associated with endothelial dysfunction in human CM [76,77]. Similarly, van der Heyde and his group demonstrated that low NO bioavailability was associated with murine CM [78,79]. Interestingly, prophylaxis with inhaled NO in CM-sensitive mice significantly reduced systemic inflammation and endothelial activation by lowering TNF-α, IFN-γ, monocyte chemotactic protein-1 (MCP-1), sICAM-1 and von Willebrand factor, and by increasing Ang-1 levels in peripheral blood [80]. The protective effect of exogenous NO on mouse CM appears associated with decreased brain vascular expression of inflammatory markers, resulting in attenuated endothelial junction damage and facilitating blood flow [81]. Lastly, treatment with exogenous L-arginine, the substrate for NOS, recently proved to be safe in a pilot study on CM patients, although effective doses still need to be optimized [82].

Furthermore, during malaria infection both host and parasite undergo strong oxidative stress, which leads to increased production of reactive oxygen species (ROS) and subsequent protein and lipid peroxidation [83,84]. The coexistence of both parasite and erythrocyte is a matter of a delicate balance: low ROS concentrations seem to inhibit parasite growth, whereas larger amounts may damage vascular endothelial cells and increase vascular permeability [85]. Oxidative stress paradoxically has both a pathogenic and protective role in CM [86]. An anti-oxidant diet was shown to reduce BBB damage and counteract CM development in CM-sensitive mice [87], and anti-oxidant adjuvant therapy, provided at the initial stages of murine CM, prevented the development of persistent cognitive damage [88]. In contrast, NADPH-deficient mice were shown to develop CM despite the lack of ROS production, suggesting that ROS did not contribute to CM pathogenesis [89,90]. To reconcile such an apparent inconsistency, Linares and colleagues have recently shown that glutathione peroxidase and heme oxygenase-1 up-regulation cooperate to suppress superoxide dismutase, catalase, heat shock protein-70 and thioredoxin-1 down-regulation effects in murine CM, counteracting oxidative damage and maintaining redox equilibrium [91]. In human CM, ROS have been associated with a pathogenic role thus far. *In vitro*, ROS inhibition was shown to protect brain endothelial cells against *P. falciparum*-induced apoptosis and to decrease iRBC cytoadherence through ICAM-1 down-regulation and iNOS induction [92,93]. Consistently, in a recent clinical study performed on fifty Indian children with severe malaria, oxidative stress was associated with disease severity [94].

### Blood–brain barrier impairment in cerebral malaria

**Figure 2:**
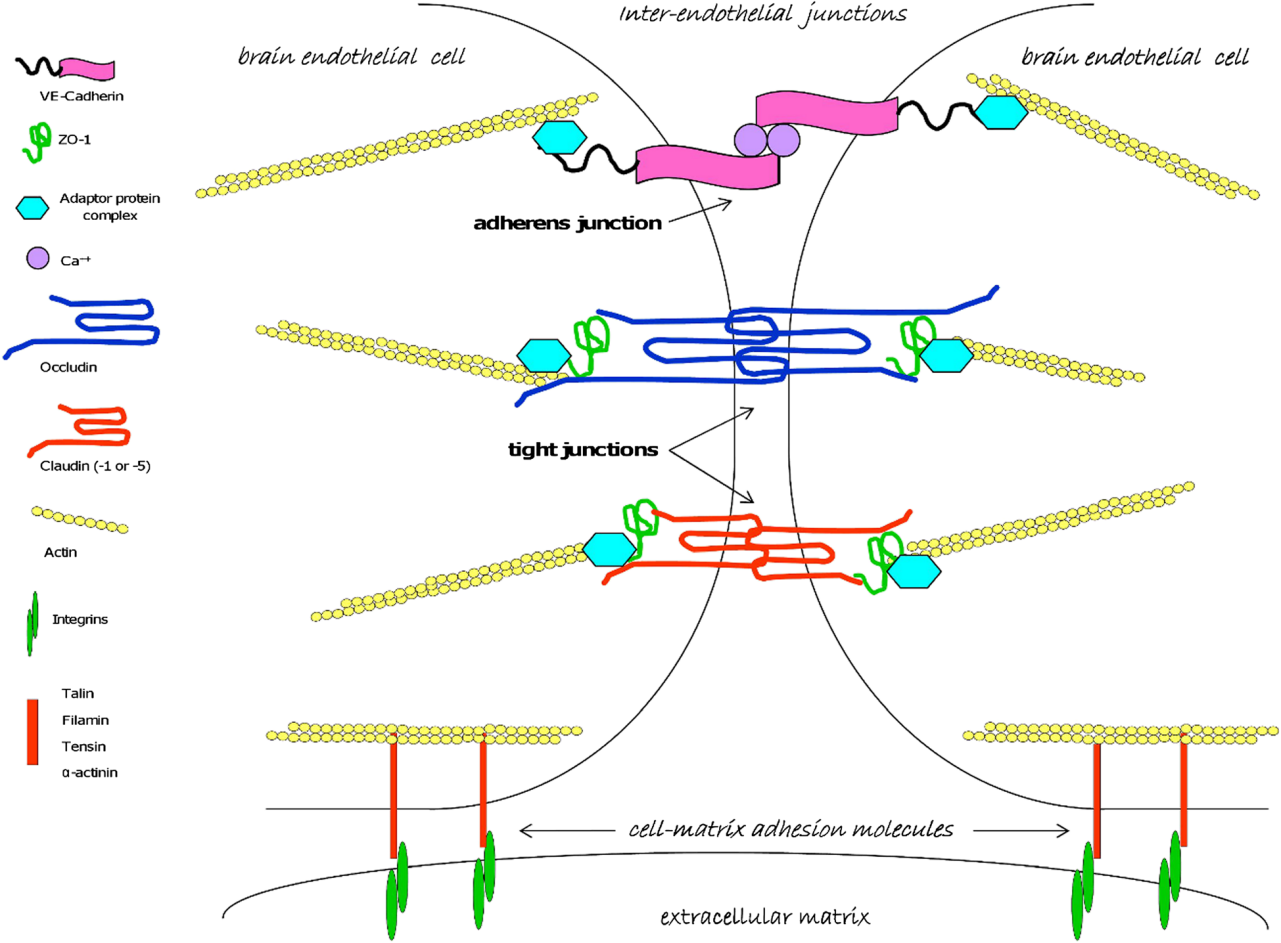
**Blood–brain barrier structure: cerebral microvascular inter-endothelial junctions (adherens and tight junctions) and cell-matrix adhesion molecules.** Diagram showing the structures of CNS inter-endothelial junctions, including adherens junctions and tight junctions, and of cell-matrix adhesion complexes, including talin, filamin, tensin or α-actinin filaments associated with integrins in the extracellular matrix. The core of adherens junctions results after the interactions among transmembrane glycoproteins, such as VE-cadherin, whose cytoplasmic face is linked to the catenin family members, including p120-catenin, β-catenin, and α-catenin. Tight junctions are composed of a branching network of sealing strands, each of which is formed by extracellular domains of transmembrane proteins, claudins and occludin, joining one another directly. These transmembrane proteins associate with different peripheral membrane proteins such as ZO-1 located on the intracellular side of plasma membrane, anchoring the strands to the actin component of the cytoskeleton.

The BBB is one of three main barrier defences protecting the CNS. It is constituted of cerebral vascular endothelial cells, which do not form a rigid structure, but rather a dynamic interface with a range of physical, biochemical and immune properties and functions, built from effective inter-cellular junctions and cell-matrix adhesion molecules, enzymes, and trans-endothelial transport systems [95]. In particular, BBB integrity is dictated by tight junctions between adjacent endothelial cells, forming a network of strands composed by several proteins, including junctional adhesion molecules, claudins (mainly -1 and -5) and occludin, which interact with cellular actin through cytoplasmic proteins such as zonula occludens-1 (ZO-1) [96]. Figure 2 depicts the structure of neural inter-endothelial tight junctions, along with cell-matrix adhesion complexes including talin, filamin, tensin or α-actinin filaments associated with integrins. We will next discuss how the disruption of these molecules by host proteolytic enzymes such as MMPs could play a relevant role in CM pathophysiology.

BBB functional integrity and permeability are generally assessed by evaluating the passage of molecules from the blood into the cerebral-spinal fluid (CSF). BBB permeability is determined by size and charge of the molecules, and the presence of specific BBB receptors to aid in the transport of certain molecules. The importance of BBB physiology and pathology has led to the development of several BBB models to better investigate the physiological, anatomical and functional characteristics [97]. However, once again the current experimental data on BBB status during CM are high variable among different model systems [98].

#### Phenotype of brain and non-brain endothelial cells co-cultured with Plasmodium iRBCs *in vitro*

As discussed below and summarized in Table 1, evidence showing differential phenotypes between neural and non-neural endothelial cells after co-culture with *Plasmodium* iRBCs comes from several *in vitro* studies [56,93,99-108].

**Table 1:** **Phenotype of endothelial cells after co-culture with infected red blood cells *in vitro***

**Endothelial cell type**	***Plasmodium *** **strain**	**Evaluated parameters**	**Endothelial phenotype**	**Ref.**
Porcine brain capillary endothelial cells (PBCEC)	*P. falciparum*	- ICAM-1, E-selectin expression;	- increased ICAM-1 and E-selectin	[99]
		- TEER;	- decreased BBB function;	
		- tight junction expression	- tight junction disruption	
Human umbilical vascular endothelial cells (HUVEC) co-cultured with iRBC-fed peripheral blood mononuclear cells	*P. falciparum from patients with uncomplicated malaria, severe malaria, or CM*	mRNA expression of:	- increased adhesion molecule mRNA (not CM-specific);	[100]
		- adhesion molecules (ICAM-1, VCAM-1, E-selectin);		
			- reduced tight junction mRNA (CM-specific)	
		- tight junctions (occludin, vinculin, ZO-1)		
TNF-α- or LT-α-activated human brain endothelial cell line (HBEC-5i) (with/without platelet co-culture)	*P. falciparum*	- permeability to 70-kDa dextran;	- increased BBB permeability;	[101]
		- TEER;	- decreased BBB function;	
		- endothelial microparticle release;	- increased microparticle release;	
		- endothelial apoptosis	- increased endothelial apoptosis (all effects potentiated by platelets)	
Human brain microvascular endothelial cells (HBMEC); HUVEC	*P. falciparum*	- ICAM-1 expression	increased ICAM-1 expression in HBMEC but not in HUVEC	[93]
HBMEC	*P. falciparum*	- electrical cell substrate sensing;	- reduced BBB function;	[102]
		- TEER	- increased BBB permeability	
Human dermal microvascular endothelial cells (HDMEC); human lung microvascular endothelial cells (HLMEC) (with parasite sonicates or iRBCs)	*P. falciparum*	- immunofluorescence staining of ZO-1, claudin-5, VE-cadherin;	- loss in total protein content of claudin-5;	[103]
		- observation of inter-endothelial gaps in monolayers;	- redistribution of ZO-1 from cytoskeleton to membrane and cytosolic/nuclear fractions;	
		- evaluation of pro-inflammatory response, direct cellular cytotoxicity or cell death.		
			- minimal inflammation and death (all effects only with sonicates)	
HBMEC	*P. falciparum*	- expression of transcriptome (including ICAM-1 and pro-inflammatory molecules)	- increased expression of ICAM-1 and pro-inflammatory molecules	[104]
HBEC-5i; immortalized human cerebral microvascular cell line hCMEC/D3	*P. falciparum*	- immunofluorescent microscopy to evaluate malaria antigen presentation by endothelial cells;	- malaria antigen presentation by endothelial cells;	[56]
			- tight junction opening;	
		- TEER	- increased BBB permeability	
hCMEC/D3	*P. falciparum*	- fluorescent permeability assay;	- increased BBB permeability;	[105]
		- expression of cell adhesion molecules and tight junctions	- increased ICAM-1 expression;	
			- cytoadherence;	
			- altered ZO-1 distribution	
TNF-α-activated subcutaneous fat tissue-derived EC from patients with uncomplicated malaria or CM	*P. falciparum*	- adhesion molecule expression (ICAM-1, VCAM-1, CD61, CD62-E)	- higher ICAM-1, VCAM-1, CD61;	[106]
			- enhanced microparticle release;	
		- microparticle production;		
			- induced MCP-1 and IL-6 release;	
		- MCP-1, RANTES, IL-6 release ;	- higher caspase-3 activation (all effects CM-specific)	
		- caspase-3 activation		
HBEC-5i	*P. falciparum* (various strains)	parasite strain selection assay based on cytoadherence	CM-associated cytoadherence	[107]
Murine brain vascular endothelial cells (MBVEC) murine lung vascular endothelial cells (MLVEC)	*P. berghei* ANKA (CM model); P. berghei K173 (non-CM model)	- study of cytoadherence mechanisms;	higher VCAM-1-mediated cytoadherence in CM model compared to non-CM model	[108]

First, the effects of *P. falciparum* infection were investigated in a BBB model of cultured primary porcine brain capillary endothelial cells (PBCECs) [99]. In this study, membrane-associated malaria antigens obtained from lysed *P. falciparum* schizont-iRBCs increased endothelial E-selectin and ICAM-1 expression, reduced the trans-endothelial electrical resistance (TEER), and promoted the disruption of tight junctions, indicative of increased BBB permeability.

Consistently in various types of human brain endothelium, including HMBEC primary cultures and HBEC-5i or hCMEC/D3 cell lines, iRBCs were also shown to increase ICAM-1 expression [93,99,104,105], to reduce TEER [56,101,102], to alter tight junction expression and distribution [56,105], and to enhance BBB permeability to 70-kDa dextran [101]. Interestingly, platelets were suggested to play a key role in iRBC-dependent increase in BBB permeability, releasing microparticles and causing cell apoptosis in TNF-α- and LT-α-activated HBEC-5i [101]. In hCMEC/D3 cells, iRBC-increased cell adhesion and paracellular permeability correlated with ZO-1 disorganization, but the latter effect appeared mediated by parasite-induced metabolic acidosis, independent from cytoadherence [105]. Moreover, differential global gene expression in HBMEC after interacting with iRBCs revealed significantly up-regulated transcripts related to immune and inflammatory responses, apoptosis, cell-cell signaling, signal transduction and nuclear factor-kB (NF-kB)-activation cascade [104]. After co-culturing with iRBCs, the mRNA expression of neural endothelial pro-inflammatory chemokines (IL-6, CXCL-8/IL-8, CXCL-1/GRO-α, CXCL-2/MIP-2α, and CCL-20/MIP-3α) increased more than 100-fold, highlighting the strong inflammatory component and the active role of the endothelium in CM pathogenesis [104]. Furthermore, in TNF-α-activated subcutaneous fat tissue-derived endothelial cells, a model comparable to cerebral endothelium, *P. falciparum* iRBCs induced several CM-specific effects, including up-regulation of ICAM-1, VCAM-1, and CD61, enhancement of microparticle, MCP-1 and IL-6 release, and higher caspase-3 activation [106]. Increased levels of inflammatory cytokines may have direct systemic effects and adversely affect the clinical outcome by increasing the cytoadherence of infected RBCs to venular endothelium through up-regulation of adhesion molecules, such as ICAM-1 [93].

To assess the specificity of these effects for human cerebral endothelium, additional comparative studies were also performed using non-neural endothelial cells. Interestingly, *P. falciparum* iRBCs did not affect the expression and distribution of tight junctions (as measured by claudin-5 and ZO-1) and did not induce pro-inflammatory response or cell death in human dermal or lung microvascular endothelium, although parasite sonicates did [103]. Additionally, the up-regulating effects of iRBCs on ICAM-1 expression observed in HBMEC were not reproduced in human umbilical vascular endothelial cells (HUVEC) from healthy donors [93]. An increase in ICAM-1, VCAM-1, and E-selectin mRNA was found in HUVEC from patients with different degrees of malaria (uncomplicated, severe, or CM) after co-culturing with iRBC-fed mononuclear cells, however such increase did not appear specific for CM. On the contrary, reduced mRNA levels of tight junction proteins (occludin, vinculin, and ZO-1) were strictly associated with CM [100].

Genetic differences between *Plasmodium* strains might also play a role in CM development. Indeed, it has been shown that different strains of *P. falciparum* display variable degrees of cytoadherence to HBEC-5i [107]. Additionally, *P. berghei* ANKA, a murine CM-associated *Plasmodium* strain, induces a higher VCAM-1-mediated cytoadherence compared to *P. berghei* K173 (non-CM strain) in either brain or lung mouse vascular endothelial cells [108].

#### Blood–brain barrier and *in vivo* animal models of cerebral malaria

Several *in vivo* animal models have reported alterations in BBB after exposure to *Plasmodium* parasites or malaria products such as hemozoin (Hz, malarial pigment) [47,109-124]. As summarized in Table 2 and described below, these studies provide insightful findings regarding BBB breakdown in animal CM models.

**Table 2:** Evidence of blood–brain barrier (BBB) impairment in animal models with cerebral malaria (CM)

**Animal source**	**Plasmodium strain**	**Method to evaluate BBB integrity**	**Degree of impairment**	**Reference**
Rhesus monkey *(Macaca mulatta)*	*P. knowlesi*	Examination of movement of proteins across the BBB by radiometric and fluorimetric methods	Increase of BBB permeability	[109-111]
Rhesus monkey *(Macaca mulatta)*	*P. fragile*	Electron microscopy, immunohistochemical analysis (CD36, thrombospondin, ICAM-1), formation of rosettes	Parasitized red blood cells sequestration and adherence to endothelial cells in the cerebral microvessels, neurological symptoms similar to humans	[112]
Rhesus monkey *(Macaca mulatta)*	*P. coatneyi*	Clinical observation	Anemia, coagulopathy, and renal and metabolic dysfunction	[113]
Rhesus monkey *(Macaca mulatta)*	*P. coatneyi*	Tissue samples from the brain (cortex and white matter of the cerebrum, cerebellum, and midbrain) collected for quantitation of mRNA expression of cytokines, adhesion molecules, and iNOS	Expression of pro-inflammatory and T helper-1 cytokines, adhesion molecules, and iNOS appears to predominate in the cerebellum of infected rhesus monkeys	[114]
A/J and CBA/H mice	*P. berghei* (ANKA)	Detection of the movement of the dye Evans blue, radioisotope labelled albumin and erythrocytes	Breakdown of BBB	[115]
mouse	*P. berghei* (K173)	Histochemical and histological evaluation of cerebral lesions and their distribution	Progressive deterioration of BBB integrity	[116-118]
CBA/T6, Balb/c and DBA/2 J mice	*P. berghei* (ANKA and K173)	Evaluation of neurological signs (ataxia, hemiplegia and coma)	Increased permeability of BBB	[119]
Mouse	*P. berghei* (ANKA)	Multimodal magnetic resonance techniques (imaging, diffusion, perfusion, angiography, spectroscopy).	BBB breakdown	[120]
CM- resistant BALB/c mice	*P. berghei* (ANKA)	Evaluation of pro-inflammatory cytokines produced	BBB breakdown	[121]
C57BL/6 and BALB/c mice	*P. berghei* (NK65)	Histopathological analysis of cerebral tissue	Increased permeability of BBB	[122]
TNF-α-and LT-α-deficient mice	*P. berghei* (ANKA)	Histochemical and histological evaluation	Neurological signs of CM, associated with perivascular brain haemorrhage in TNF-α -/- mice; completely resistant to CM in LT-α -/- mice	[47]
Mouse	*P. berghei* (ANKA)	Examination of the outcome of TGF-β and TNF-α production in the context of splenocyte apoptosis	Critical balance between TGF-β and TNF-α might have a key role in BBB breakdown	[123]
Different murine models: CBA/CaJ and Swiss Webster mice (CM sensitive), Balb/c and A/J mice (CM resistant)	*P. berghei* (ANKA) *P. yoelii* (17XL) *P. berghei* (NK65) and *P. yoelii* (YM)	Examination of histopathological alterations, BBB dysfunction, or neurological signs	CM related to the opening of paracellular-junctional and transcellular-vesicular fluid transport pathways at the neuroimmunological BBB	[124]

The first animal studies on BBB permeability in malaria date back to 1968, when Migasena and Maegraith demonstrated the movement of albumin across the BBB in *Macaca mulatta* monkeys infected with *P. knowlesi*[109-111]. However, *P. knowlesi* does not induce CM. As such, the rhesus monkey infected with primate malaria parasites, *P. coatneyi* and *P. fragile*, is considered to be a more valid primate model to study in the context of severe malaria with cerebral involvement [112-114].

Of the four species of rodent malaria parasites (*P. berghei*, *P. yoelii*, *P. chabaudi*, *P. vinckei*), only a few *P. berghei* strains can induce experimental CM in mice, with the ANKA strain being the most widely studied. Symptoms of experimental CM in *P. berghei* ANKA-infected susceptible mice include paralysis, ataxia, head deviation, convulsion and coma [98]. In *P. berghei* K173-infected mice an excessive movement of water, albumin and other proteins into the brain, as well as severe brain edema, microthrombosis, sludging of mononuclear cells, arteriolar spasms, scattered disturbances of the microcirculation, and occasional proliferation of gliocytes were observed, suggesting a progressive deterioration of BBB integrity culminating in endothelial lesions and haemorrhages [31,115-118]. Of note, mouse CM models present neurological signs (ataxia, hemiplegia and coma) similar to the clinical features reported in human CM [119].

In a recent work, Penet and colleagues presented the first *in vivo* magnetic resonance study of mouse CM, demonstrating BBB breakdown in CM. Multimodal magnetic resonance neuroimaging techniques (imaging, diffusion, perfusion, angiography, spectroscopy) of *P. berghei* ANKA-infected mice revealed vascular damage, including BBB disruption and haemorrhages, major edema formation, reduced brain perfusion and ischemic metabolic profile, with reduced high-energy phosphates and enhanced brain lactate. These data strongly point to the coexistence of inflammatory response and ischemic lesions [120].

Other recent works illustrated a complex strain-dependent relationship between leukocyte recruitment, BBB permeability and chemokine production. Major pathological consequences of malaria arise from inappropriate or excessive immune response mounted by the host in an attempt to eliminate the parasite. In *P. berghei* ANKA-infected mice, inflammation of the cerebral microvasculature and leukocyte recruitment were clearly evident and found to be driven by production of pro-inflammatory cytokines (IL-12, IFN-γ) and CM development [121]. On the other hand, *P. berghei* NK65-infected mice showed enhanced production of LT-α and several chemokines (CXCL-9/MIG, CCL-2/MCP-1, CCL-3/MIP-1α and CCL-5/RANTES), but no neurological symptoms [47,122]. A complementary study performed on the same model proposed a concurrent role for Transforming Growth Factor-β (TGF-β) and TNF-α in promoting splenocyte apoptosis [123].

It should be noted that the cerebral microvascular tree contains two functionally distinct BBB: i) the physiological BBB, formed by capillaries 4–8 mm in diameter, consisting of a single layer of endothelia, gliovascular membrane, and astrocyte endfeet; and ii) the neuroimmunological BBB, formed by postcapillary venules 10–60 mm in diameter and encompassing two layers - the endothelium with its basement membrane and the glia limitans with associated astrocyte endfeet - separated by the perivascular space [125]. The physiological BBB serves as a tight diffusion barrier for small solutes while the neuroimmunological BBB permits transport of macromolecules and diapedesis of immune cells [125]. In a very recent study comparing different mouse models of experimental CM (*P. berghei* ANKA infection), human CM-like histopathology (*P. yoelii* 17XL) and non-CM (*P. berghei* NK65 and *P. yoelii* YM), Nacer and colleagues observed that the physiological BBB in the experimental CM model remained intact, whereas regulated fluid transport across the neuroimmunological BBB led to brain swelling, intracranial hypertension, coma, and ultimately death due to dysfunction of respiratory centers in pons and the medulla oblongata as a result of brain stem compression [124]. Thus, they proposed that CM may occur in two steps: 1) induction of coma based on regulated, preventable and reversible opening of the neuroimmunological BBB; and 2) endothelial death-associated haemorrhaging, which is difficult to reverse by treatment and eventually fatal [124]. A similar mechanism for neuroimmunological BBB opening in human CM would explain the reversibility of coma with treatment, the scarce traces of tissue necrosis in surviving patients, and the different neurological outcomes of patients despite similar clinical presentation [6,8,9,13,102].

#### Blood–brain barrier and human studies on cerebral malaria

BBB functional impairment during human CM has been investigated in several clinical and *post mortem* studies [126-143]. Table 3 summarizes the most relevant results. Here, the investigations on human CM patients were performed using albumin CSF/serum ratio as an indicator of BBB integrity [126-128], by *post mortem* immunohistochemical analysis [129-135], or through brain imaging techniques [136-144]. Interestingly, the BBB seems to be more impaired in children than in adults. Moreover, it appears that African and Asian patients display a different degree of BBB damage, with BBB breakdown being more likely to occur in African than Asian populations.

**Table 3:** Evidence of blood–brain barrier (BBB) impairment in human cerebral malaria (CM) patients

**Group type**	***Plasmodium *** **strain**	**Number of patients per cohort**	**Method to evaluate BBB integrity**	**Degree of impairment**	**Reference**
Thai patients	*P. falciparum*	157	Albumin CSF/serum ratio	BBB intact	[126]
Vietnamese patients	*P. falciparum*	20	Albumin and Immunoglobulins G plasma/CSF ratios	Minimal BBB breakdown in a few cases of CM	[127]
Zairean children	*P. falciparum*	21	Albumin CSF/serum ratio	BBB not impaired	[128]
Malawian children	*P. falciparum*	72	Immunohistochemistry on autopsy brain tissues	Disruption of endothelial intercellular junctions and impaired BBB function	[129]
Kenyan children	*P. falciparum*	100	Protein and immunoglobulin CSF/serum ratio	Mild BBB impairment in some cases	[130]
Malawian children	*P. falciparum*	50	Immunohistochemistry on autopsy brain tissues	BBB breakdown	[131]
Nigerian children	*P. falciparum*	61	Examination of the possible risk factors for poor prognosis and studies on *post mortem* samples	Cerebral edema and raised intracranial pressure in 50%	[132]
Thai and Vietnamese children	*P. falciparum*	65	Studies on *post mortem* samples	Cerebral sequestration of P. falciparum-infected erythrocytes	[133]
Vietnamese patients	*P. falciparum*	20	Studies on *post mortem* samples	Heterogeneous cerebral edema and plasma protein leakage	[134]
Vietnamese adults and Malawian children	*P. falciparum*	14	Immunohistochemistry	Alteration of cell junction proteins occludin, vinculin and ZO-1	[135]
Kenyan children	*P. falciparum*	14	Computed tomography	Cerebral edema and ischemia	[136]
French adults back from Cameroon, Niger, and Thailand	*P. falciparum*	3	Magnetic resonance	Hemorrhagic cortical lesions	[137]
Malian children	*P. falciparum*	8	Computed tomography	Diffuse atrophy with asymmetrical ventricle dilation, suggesting limited CSF circulation	[138]
French adult back from Equatorial Guinea	*P. falciparum*	1	Magnetic resonance	BBB breakdown	[140]
Malawian children	*P. falciparum*	14	Computed tomography	Fatal CM: cerebral edema, large vessel infarcts; Non fatal CM with neurological sequelae: focal/multifocal atrophy	[141]
Indian adults	*P. falciparum*	4	Magnetic resonance	Bithalamic infarctions with or without haemorrages	[142]
Malawian children	*P. falciparum*	120	Magnetic resonance	increased brain volume; abnormalities in cortical, deep gray, and white matter structures	[143]
Malawian children	*P. falciparum*	38	Magnetic resonance	periventricular and subcorical T2 signal changes, atrophy, and focal cortical defects	

One of the first studies on Asian patients was conducted in Thailand [126]. In this work, albumin CSF/serum ratios were higher in CM patients than in controls, but it did not correlate with coma and mortality. Thus, the authors concluded that their data did not support the idea that cerebral edema might be the cause of coma. More than a decade later, albumin and Immunoglobulins G plasma/CSF ratios were found to be only mildly impaired in Vietnamese patients, suggesting only minimal degree of BBB breakdown in few CM cases [127]. Therein, human CM appeared to cause only subtle functional changes in BBB integrity, with minimal intra-parenchymal inflammatory response compared with other neurologic infections, such as cryptococcal, tubercular, and acute bacterial meningitis [127].

Regarding African populations, a study on Zairean children showed no difference in CSF albumin compared to controls [128]. However, in Malawian children with CM, the activation of endothelial cells and macrophages, along with the disruption of endothelial intercellular junctions in vessels containing sequestered iRBCs, and subtle but measurable changes in albumin CSF *versus* albumin serum levels were observed. Nevertheless, negligible leakage of plasma proteins was still apparent [129]. In Kenyan children with CM, protein and amino acid levels in paired plasma and CSF samples were measured, showing that BBB was mildly impaired in some children with severe *falciparum* malaria [130]. However, this impairment was not confined to CM, as it was also reported in children with prostration-associated malaria and, to a lesser extent, in children with malaria and seizures. Evidence of intrathecal immunoglobulin synthesis in children with malaria was also observed [130]. Finally, data obtained in a recent work performed on Malawian children are consistent with the proposed link between iRBCs sequestration and intravascular/perivascular pathology in fatal pediatric CM, resulting in myelin damage, axonal injury, and BBB breakdown; however, no Hz-laden monocyte extravasation was found [131].

Pathological studies on *post mortem* samples of CM patients showed cerebral edema and raised intracranial pressure in 50% of West African children [132] but not in South Asian adults [133,134] or Malawian children [129]. Nevertheless, an important correlation between sequestration of iRBCs in the brain microvessels and the malaria-related encephalopathy was shown in Asian patients [133]. The adhesion of iRBCs to brain microvessels is mediated by specific receptors on the host endothelium, including ICAM-1, CD36 and CD31 [22-25]. Immunohistochemistry showed altered distribution of the cell junction proteins occludin, vinculin and ZO-1 in Vietnamese adults and Malawian children with CM [129,135]. Sequestration of iRBCs in cerebral microvessels was significantly higher in the brains of patients with CM compared with non-CM patients in all parts of the brain (cerebrum, cerebellum, and medulla oblongata), and was quantitatively associated with *pre mortem* coma [129].

In recent years, several imaging studies have been also conducted on the brains of CM patients during disease progress or after recovery [136-144]. Using magnetic resonance or computed tomography, several common features implicating BBB damage have been observed, including cerebral edema, increased brain volume, ischemia and large vessel infarcts, hemorrhagic cortical lesions, focal and multifocal atrophy, and limited CSF circulation [136-139,141-144]. Interestingly, magnetic resonance in a recently published case-report of a 37-year-old French patient with malaria travelling back from Equatorial Guinea, showed that he developed posterior reversible encephalopathy syndrome, which is characterized by diffuse symmetric signal-intensity abnormalities of white matter in the posterior circulation territory [140]. Since data from previous literature suggest one of the mechanisms of posterior reversible encephalopathy involves capillary leakage and acute disruption of the BBB, the authors concluded that this case-report supports the theory of BBB disruption as a key factor for CM development [140].

#### Blood–brain barrier impairment in cerebral malaria: some reflections upon the available studies

Clearly there is much discrepancy on the extent of BBB damage between animal and human models of CM. While several studies performed on mouse CM models suggest a strong BBB breakdown [115-124], data on increased BBB permeability in human CM are somehow less evident, generally suggesting the occurrence of only mild BBB impairment, characterized by a relevant degree of tight junction disruption, but lacking molecule exchange between serum and CSF [126-135].

In this context, it should be noted that the relevance of murine CM models for studying CM pathophysiology has been a topic of big debate in the recent years [49]. Being clearly an inflammatory syndrome with local vascular endothelial activation, murine CM displays obvious differences and some similarities to the clinical and pathological features of human CM, such as signs of vascular inflammation/damage [145]. A recurring issue concerns the degree of iRBC sequestration in the brain and other organs of *P. berghei* ANKA–infected mice. Although recent data find increased iRBC accumulation during murine CM in multiple organs including the brain [146], *P. berghei* infection is generally acknowledged to promote marked accumulation of leukocytes (particularly monocyte, macrophages and T cells), which is in stark contrast to human CM [147]. Thus, despite several processes shared either by murine or human CM, the changes in the endothelial cell microenvironment induced by cytoadherence and inflammation are not the same [104]. Additionally, mouse studies suggesting associations between high levels of cytokines and CM [58-61] have been recently challenged by works showing that high levels of pro-inflammatory cytokines such as TNF-α are poor indicators of human CM in African children [64-67]. Thus, future experimental studies on alternative animal models (non-human primates and other mouse models) are encouraged and urgently necessary to better understand the pathological processes underlying human infection [49].

Another interesting point emerging from clinical data is that the BBB appears more impaired in children than in adults [126-143]. Since CM often strikes children at a critical time in brain development, Hawkes and colleagues have nicely hypothesized that developmental changes in the cerebral vasculature may account for some of the differences in disease presentation and outcome between children and adults, including mortality, seizures and neurocognitive sequelae, rates of associated anemia and renal dysfunction, retinal vessel changes, frequency of ring haemorrhages, and inflammatory cell accumulation in brain microvessels [148]. To avoid any misunderstanding, it has been pointed out that the widespread belief amongst neurotoxicologists that BBB is immature or even absent in the newborn is contrasted by a substantial body of evidence supporting the concept of well-developed barrier mechanisms in the developing brain [149,150]. For instance, inter-cellular tight junctions between cerebral endothelial cells and between choroid plexus epithelial cells are functionally effective as soon as they differentiate [150]. Nevertheless, the brain develops within an environment that is different from that of the rest of the body, and the developing brain possesses a number of unique features not generally present in the adult [149]. Interestingly, certain genes coding for influx/efflux proteins are expressed at much higher levels early in development than in the adult, and there is physiological evidence that these transport systems are functionally more active in the developing brain [150]. How such differences between the pediatric and adult BBB can affect CM pathogenesis and correlate with enhanced BBB permeability in pediatric CM is still unknown. Future research aimed at shedding light on this topic will certainly be useful.

### Involvement of matrix metalloproteinases in cerebral malaria

In the last decade, experimental evidence implicated a specific family of host proteolytic enzymes known as MMPs in malaria pathogenesis [2,151-154]. MMPs are either secreted or membrane-bound zinc-dependent proteases, and their role is also related to the inflammatory response and the BBB function [155-163]. Members of the MMP family are produced by a broad spectrum of specialized cells, including fibroblasts, endothelial cells, lymphocytes, monocytes, macrophages, smooth muscle cells, glial cells, and neurons [155,158].

**Figure 3:**
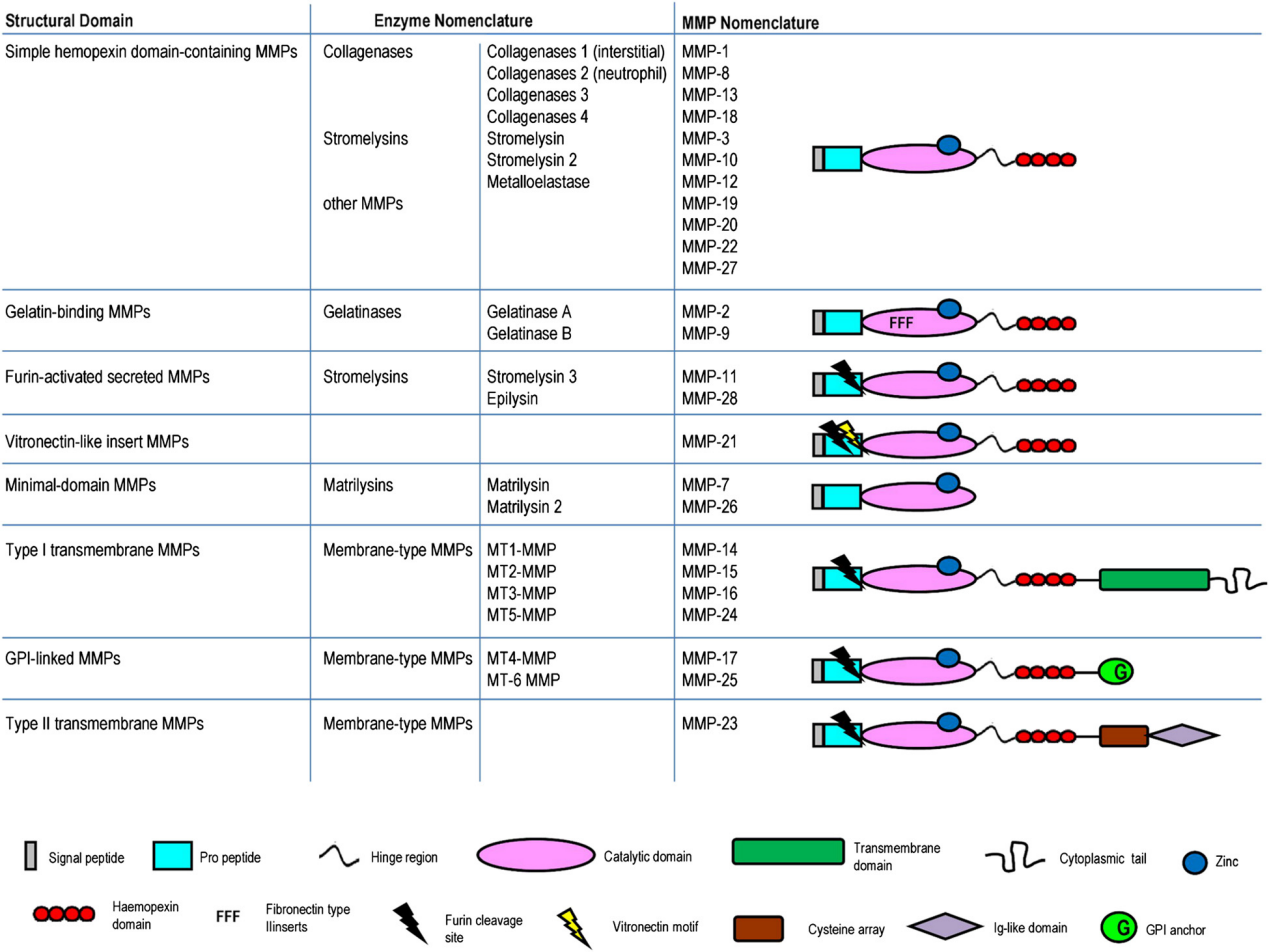
**Nomenclature and structure of mammalian matrix metalloproteinases(MMPs).** The mammalian MMP family encompasses 25 members, categorized by different numbers (standard MMP nomenclature) or named depending on their matrix substrates (enzyme nomenclature). Each MMP displays some conserved structural domains, including: i) an N-terminal signal peptide required for secretion; ii) a cleavable pro-domain maintaining enzymatic latency; iii) a catalytic and Zn-binding domain; and iv) a C-terminal hemopexin domain. Optional MMP motifs include a fibronectin-type domain, a vitronectin motif, a furine cleavage site, three head-to-tail cysteine-rich repeats, and (for MT-MMPs) a C-terminal transmembrane domain or GPI anchor occasionally associated with a cytoplasmic domain.

As detailed in Figure 3, the mammalian MMP family encompasses 25 members, categorized by different numbers or named depending on their matrix substrates. MMPs are evolutionarily conserved and tightly regulated. Conserved protein domains include an N-terminal signal peptide required for secretion, a cleavable pro-domain maintaining enzymatic latency, a catalytic domain, a Zn-binding domain, and (besides the minimal MMPs) a C-terminal hemopexin domain thought to be important for protein-protein interactions [155,157,160,162,163]. The active domain and the Zn-binding domain are essential for catalytic activity: upon pro-domain cleavage a Zn^2+^-ion becomes available to coordinate with a hydrolytic water molecule to enable nucleophilic attack of a substrate, and the enzyme is functionally active [164]. Additional MMP motifs include a gelatin-binding fibronectin-like domain, a serine-, threonine- and proline-rich collagen type V-like domain, a C-terminal transmembrane (TM) domain or GPI anchor, and in some cases a cytoplasmic domain [155,157,160,162,163]. MMP-2 and MMP-9 (also named gelatinase A and B) are further characterized by the presence of three head-to-tail cysteine-rich repeats within the catalytic domain reminiscent of the collagen-binding type II repeats of fibronectin and this domain is necessary for the binding and cleaving activities of these MMPs [161,165,166]. Six membrane-anchored MMPs (MT1- to MT6-MMPs) have a basic RX(K/R)R motif at the C-terminal end of their pro-domains. This motif is recognized and cleaved intracellularly by furin-like proteases. Four MT-MMPs (MT1- to MT4-MMPs) are anchored to the cell membrane through a type I TM domain while the other two MT-MMPs (MT5- and MT6-MMPs) are tethered to the membrane via a GPI moiety. An additional MT-MMP (MMP-23) has an N-terminal type II TM domain [162].

MMPs are regulated at multiple levels including transcription, translation, compartmentalization, secretion, activation, and inhibition by protein inhibitors. Most MMPs are found at low levels and not constitutively transcribed, but are expressed after external induction by pro-inflammatory molecules, growth factors, NO, cell-cell interactions, cell-matrix interactions, UV radiations [155,157,159-162]. Several signalling pathways and transcription factors are known to regulate MMP expression, including mitogen-activated protein kinases (MAPKs), NF-kB, and activator protein-1 (AP-1) [155,165,167]. After synthesis, MMPs are stored in inflammatory cell granules, which restrict their action [161]. Furthermore, MMPs are produced as inactive zymogens, referred to as pro-MMPs. Activation is achieved by various proteases (other activated MMPs and several serine proteases) or ROS that disrupt the interaction between the active site zinc atom in the catalytic domain and the conserved cysteine within the pro-domain. Pro-MMPs can be cleaved and activated through different mechanisms and in a context-specific manner. For example, pro-MMP-9 is activated through a proteolytic cascade sequentially involving plasminogen, MMP-3 and MMP-1 [165]. The activation of proMMP-2 requires previous formation of a pro-MMP-2/tissue inhibitor of metalloproteinase-2 (TIMP-2)/MT1-MMP (MMP-14) multimeric complex [166]. In addition to pro-MMP-2 activation, the binding of TIMP-2 to MT-1-MMP and MT-3-MMP slows down the autocatalytic turnover of these MT-MMPs, paradoxically enhancing surface proteolysis further by stabilizing the pool of active enzyme at the cell surface [160,168]. Once MMPs have been released into the extracellular space or anchored to the membrane and activated, they are kept in check by their endogenous tissue inhibitors (four different forms, from TIMP-1 to TIMP-4). TIMPs inhibit MMP activity with relatively low selectivity in a 1:1 stoichiometric ratio. Interestingly, the ratio of MMP:TIMP can also influence activation mechanisms [157,167].

MMPs were originally discovered in tadpoles as the agents responsible for tail resorption during frog metamorphosis. Thus, they were first characterized as proteases involved in degrading structural proteins comprising the extracellular matrix (ECM) and sub-endothelial basement membranes [155]. However, MMPs are now known to have more sophisticated processes than mere ECM turnover. MMPs can also cleave a growing spectrum of other substrates, including cytokines, chemokines, growth factors, hormones, chemotactic and adhesion molecules, membrane receptors, intercellular junctions, as well as other proteases, including some hemostasis-related molecules and MMPs themselves, protease inhibitors, clotting factors, and antimicrobial peptides [169-171]. MMP-dependent cleavage can serve to activate, inhibit, process, release, shed, or reveal cryptic codes in the substrates they act on. Therefore, the once formidable proteolytic potential of MMPs is now realized to serve essential roles in promoting or inhibiting cell survival, proliferation, migration, invasion, hemostasis and inflammation in either physiological or pathological processes [159,167,169,170].

In physiology, MMPs are involved in diverse biological mechanisms ranging from wound repair to pregnancy [155,157,159,167]. In pathology, MMP dysfunction has been implicated in cancer, cardio-vascular diseases, emphysema, acute renal failure, ophthalmic pathologies, neuroinflammation, neurodegenerative disorders, autoimmune diseases [156-160,167,169,170] and, very recently, malaria [2,151-154].

#### Matrix metalloproteinases and animal models

*In vivo* mouse models of CM have recently implicated MMP dysfunction in disease pathology, although it should be kept in mind that experimental CM presents important differences compared to human CM, such as leukocyte sequestration in cerebral microvessels and subsequent migration into brain parenchyma [49]. An excellent study performed by Van den Steen and his group comprehensively investigated mRNA expression levels of MMPs and protein release or pro-enzyme activation in five different organs (brain, lung, spleen, liver, and kidney) from CM-sensitive C57B1/6 mice infected with *P. berghei* ANKA (CM model) or *P. berghei* NK65 (non-CM model) and CM-resistant Balb/C mice infected with *P. berghei* ANKA (CM-resistant model) [172]. Importantly, they observed enhanced expression and activation of monocytic (CD11b+) MMP-9 in brains of CM mice [172] specific to CM, as suggested by comparison with non-CM models, such as lung pathology [173]. Additionally, tissue-specific increases in mRNA expression were found for several MMPs, including MMP-3, -4, -8, and -13 in spleen, MMP-8, -12, -13, and -14 in liver, and MMP-8 and -13 in brain. All of these increases were more pronounced in the CM model. In a CM-resistant model, MMP-3 expression was significantly enhanced, suggesting a protective role for this MMP in CM [172]. In another study, CM mice showed increased neural MMP-7 protein levels [151]. Interestingly, urokinase-type plasminogen activator (uPA) -/- or urokinase-type plasminogen activator receptor (uPAR) -/- knock-out mice with CM displayed enhanced survival and attenuated thrombocytopenia [174].

A parasite molecule, malarial pigment Hz - a lipid-bound ferriprotoporphyrin IX produced by *P. falciparum* after hemoglobin catabolism [175] - is proposed to play a role in experimental CM. Indeed, using a sensitive fluorometric method to determine Hz content in blood and tissue samples from mice infected with *P. berghei* NK65 (non-CM model) or ANKA (CM model), Sullivan and colleagues observed increased Hz levels in tissue correlating with the duration of infection, with neural Hz levels being higher in CM than non-CM mice, raising the possibility that Hz presence may be associated with cerebral pathology [176].

Interestingly, *in vitro*, Hz appears to play a major role in MMP dysfunction. Phagocytosis of Hz by RAW 264.7 rat macrophage cell line was shown to impair expression of several inflammatory molecules [177] and, after an early inhibitory peak, to increase the long-term mRNA expression of MMP-9 [178]. This effect was related to the lipid moiety of Hz, since lipid-free synthetic Hz (β-hematin) did not modulate MMP-9 expression. The Hz-dependent enhancement of MMP-9 transcription and protein release was mimicked by 4-hydroxy-2-nonenal (4-HNE) [178], a molecule generated by Hz from polyunsaturated fatty acids [179].

#### Matrix metalloproteinases and human studies

**Figure 4:**
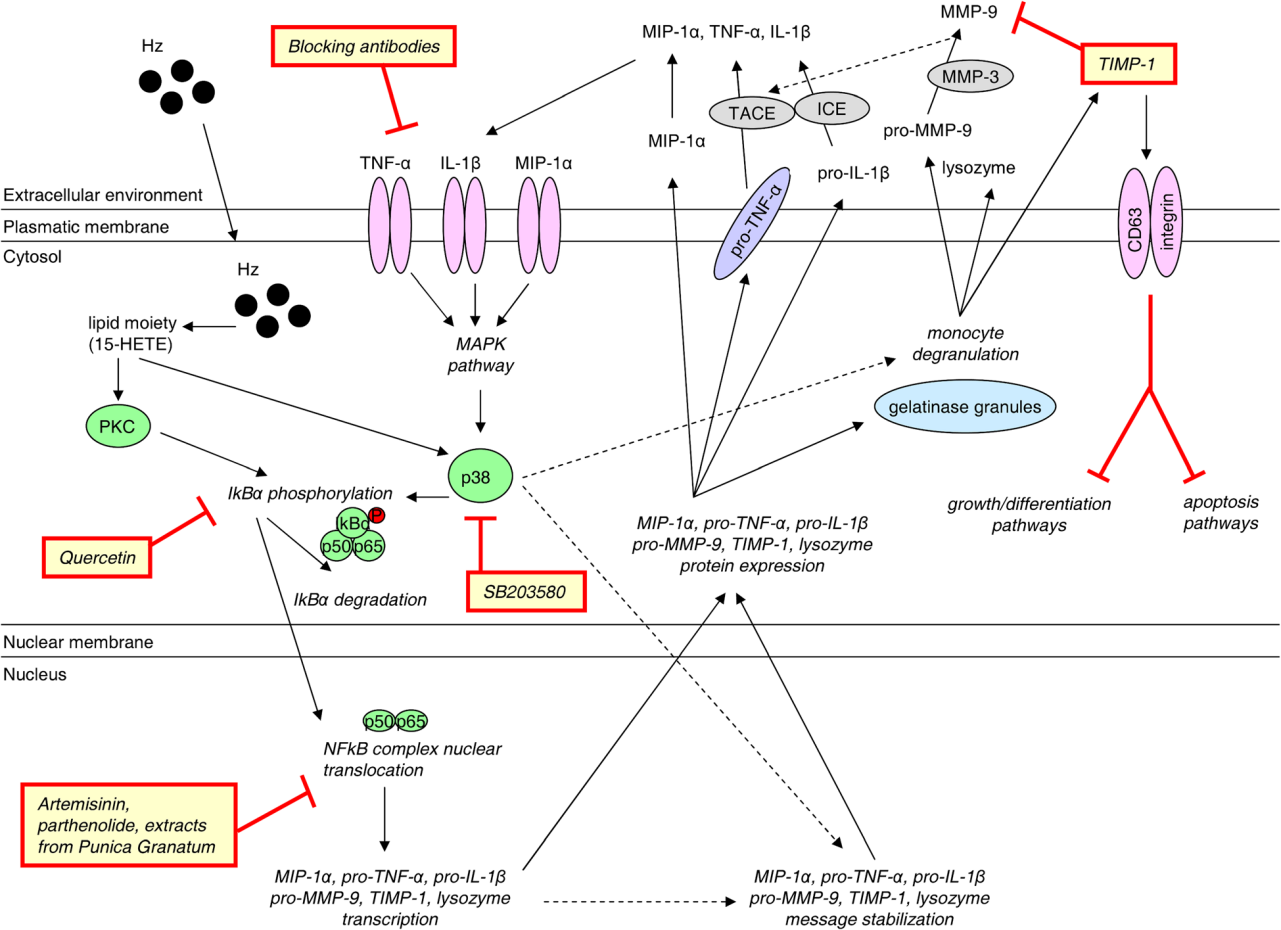
**Principal mechanisms underlying Hz-dependent dysregulation of matrix metalloproteinase-9 (MMP-9) and related molecules in human monocytes.** The most relevant Hz-dependent mechanisms underlying aberrant MMP-9 function in malaria are based on current evidence from *in vitro* models of cultured human monocytes. After Hz phagocytosis, some lipoperoxidation products generated by Hz autocatalysis such as 15-HETE can promote early and late activation of PKC and p38 MAPK. These kinases have been associated with cytosolic I-kBα phosphorylation and degradation, resulting in subsequent nuclear translocation of NF-kB p50 and p65 subunits. Consequently, the transcription and protein expression of several pro-inflammatory molecules including TNF-α, IL-1β, and MIP-1α, and of some proteolytic enzymes or inhibitors such as lysozyme, pro-MMP-9, and TIMP-1 is enhanced. p38 MAPK can also promote monocyte degranulation, releasing pro-MMP-9, TIMP-1 and lysozyme into the extracellular environment. After secretion of these molecules, several proteolytic events can occur. Active MMP-9, generated from MMP-3 processing of the pro-enzyme, can further modulate TNF-α shedding from cell membrane in a similar manner as TACE, whereas ICE activates IL-1β after cleaving its pro-peptide. Soluble TNF-α, IL-1β, and MIP-1α have been shown to play a key role in mediating Hz effects on MMP-9, lysozyme, and TIMP-1 production, possibly generating some auto-enhancing loops. Hz-enhanced MMP-9 could favour CM development through complementary proteolytic activities (see Figure 5). On the other hand, TIMP-1 is primarily referred to as a MMP-9 inhibitor, thus TIMP-1 Hz-enhanced levels could supposedly be protective. However, several MMP-independent functions such as inhibition of cell apoptosis and growth have been recently described for TIMP-1. Thus, Hz-enhanced TIMP-1 protein may play a role in prolonged survival of impaired Hz-fed monocytes, in their altered maturation to dendritic cells and in their reduced ability to coordinate erythropoiesis. Finally, enhanced plasma levels of human lysozyme have been depicted as a risk factor for severe malaria.

*In vitro* studies using human monocytes and endothelial cells [152,180-195] provide convincing and homogeneous evidence for Hz-dependent mechanisms underlying aberrant MMP-9 function (see Figure 4). In a series of works performed with human adherent or immunopurified monocytes from peripheral blood, the phagocytosis of free Hz or Hz-containing trophozoites enhanced MMP-9 mRNA levels, protein expression, and activity [180-186]. This observation was also investigated using THP-1 monocyte cell line [187]. Hz-fed monocytes display increased total gelatinolytic activity [186] and invasiveness [180] caused by MMP-9 - but not MMP-2 [188] - enhancement.

Increased MMP-9 function in human monocytes appears to be mediated by Hz-dependent over-production of several pro-inflammatory molecules, including TNF-α [180], IL-1β [181], and CCL-3/MIP-1α [184]. Further investigation revealed increases in MMP-9 [181], TNF-α [189,190] and IL-1β [181,190], but not CCL-3/MIP-1α [183,190], were dependent on the lipid moiety of Hz. These studies unveiled a major role for 15-HETE, a potent lipid peroxidation derivative generated by Hz autocatalysis. Hz was also causally related to increased TIMP-1 and lysozyme release from human adherent monocytes, two molecules stored in gelatinase granules along with MMP-9 [186,190-192]. Further studies also showed that Hz-induced monocyte degranulation was mediated by TNF-α, IL-1β and MIP-1α/CCL-3 [191,192] and dependent on Hz lipid moiety, suggesting a major role for 15-HETE [190]. The heme core of Hz was shown to bind MMP-9 hemopexin domain and to prime the activation of the zymogen by other MMPs, such as MMP-3 [193]. The mechanisms underlying Hz-dependent enhancement of MMP-9, TNF-α, IL-1β, CCL-3/MIP-1α, TIMP-1 and lysozyme appear to involve NF-kB activation, as suggested by results from parallel works performed with adherent monocytes from peripheral blood [182,186,190,191] and THP-1 cell line [187]. In these works, Hz-induced enhancement of MMP-9, TNF-α, IL-1β, CCL-3/MIP-1α and TIMP-1, as well as total gelatinolytic and lysozyme activity were abrogated by using different NF-kB inhibitors showing anti-malarial properties (quercetin, artemisinin, parthenolide, and extracts from *Punica granatum*). Moreover, results from experiments with SB203580, a known inhibitor of p38 MAPK pathway suggest that concurrent activation of p38 MAPK pathway seems to be mandatory for Hz- and 15-HETE-dependent increased MMP-9 [185] and related molecules TNF-α, IL-1β, CCL-3/MIP-1α, TIMP-1 and lysozyme [186,190,191]. On the contrary, ERK and JNK MAPK pathways do not seem to be activated by Hz [185].

Additional evidence on Hz-dependent MMP dysregulation is also derived from studies using human endothelial cells. In the human microvascular endothelial cell line HMEC-1, either free Hz [194] or Hz-containing-iRBCs [195] induced the release of pro-MMP-9 and active MMP-9 proteins *de novo* without altering pro-MMP-2 basal levels. Interestingly, Hz also enhanced the protein levels of basal MMP-1 and MMP-3, two MMPs sequentially involved in pro-MMP-9 activation. Consequently, total gelatinolytic activity and cell invasion were increased. More evidence of Hz-triggered enhancement of MMP-9 protein release emerges from studies using human endothelial cells from large calibre vessels [152]. Similar to human monocytes, a role for the Hz lipid moiety also appears likely in endothelial cells, although the molecules responsible for these effects have not yet been identified [194].

Little evidence exists on the *in vivo* involvement of MMPs in human CM from organ autopsy studies or from fluid (blood, serum) analysis of patients with uncomplicated or severe malaria [65,196-201]. The little data that does exist are somehow conflicting and still incomplete.

The accumulation of pro-angiogenic uPAR [196], MMP-1 and VEGF [197] in Durck granulomas, along with the lack of anti-angiogenic endostatin/collagen XVIII in ring haemorrhage areas [198] has been observed in the brains of patients with fatal CM, suggesting that the proMMP-9 proteolytic machinery is activated in areas of intense parasite sequestration and vascular damage. Consistently, microarray analysis performed on whole blood from Kenyan children with severe malaria showed *P. falciparum* activation of the human MMP-9 gene [199].

However, sera of Gabonese and Ghanaian children with uncomplicated or severe malaria did not display altered MMP-9 levels [65,200]. Moreover, serum MMP-8 levels were elevated in Gabonese children with either uncomplicated malaria or CM [200], but not in Nigerian children with uncomplicated malaria [201]. Nonetheless, it has been argued that serum levels might not be an ideal source for reliable data concerning MMP levels, since MMP release from blood cells during sample processing might give a highly non-specific background result, thus hindering the ability to assess true concentrations of circulating MMPs [202].

#### Possible role of matrix metalloproteinases in pathophysiology and therapy of cerebral malaria

**Figure 5:**
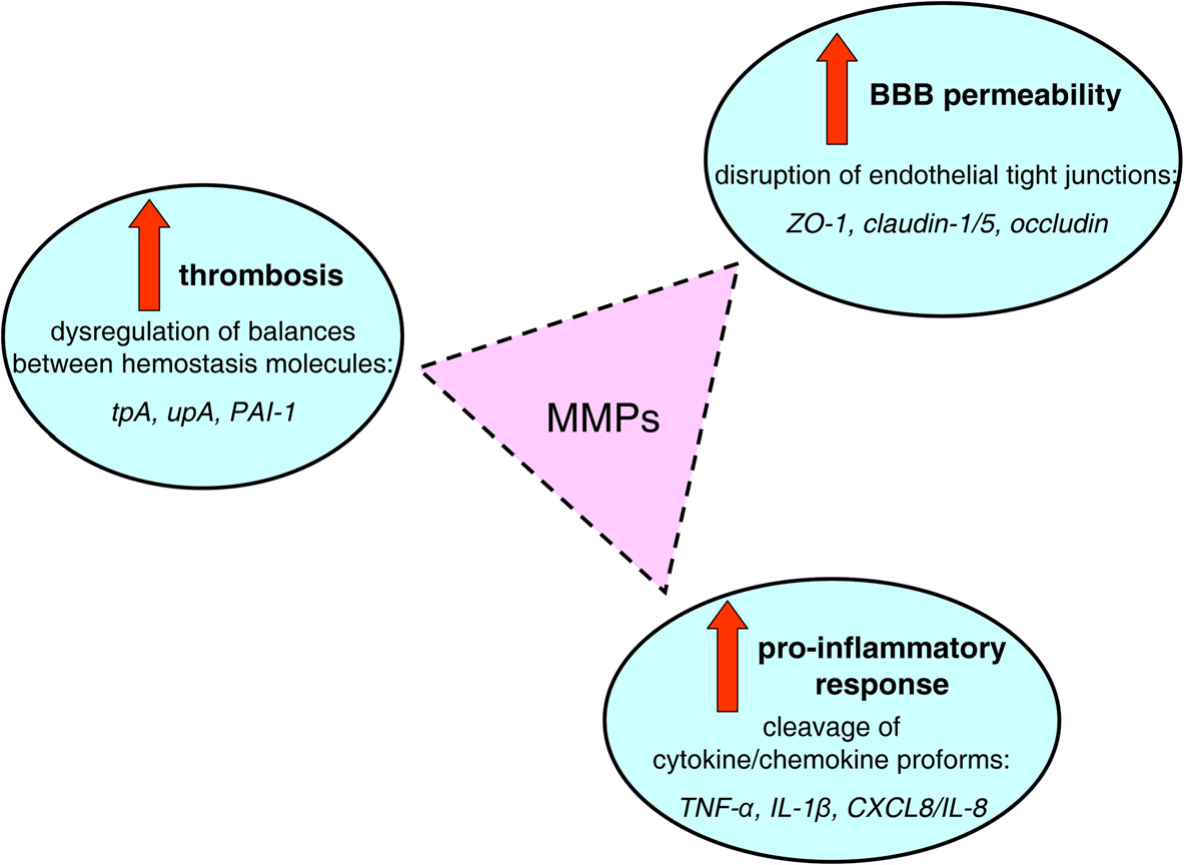
**Multiple putative roles of matrix metalloproteinases (MMPs) in cerebral malaria (CM) according to their biochemical functions.** MMPs could play an active role during CM development through several complementary mechanisms: i) by disrupting endothelial tight junctions after protein degradation of ZO-1, claudins and occludin, thus causing an increased BBB permeability; ii) by promoting TNF-α shedding, IL-1β activation and CXCL8/IL-8 potentiation after proteolytic cleavage of their pro-domains, therefore inducing exacerbated pro-inflammatory response; iii) by processing some CM-associated hemostatic factors such as tpA, upA, and PAI-1, thus increasing the risk for thrombotic events.

It is likely that MMPs play an active role in several steps during CM development as they can process a large repertoire of substrates [169-171], including pro-inflammatory molecules, tight junctions, and hemostatic factors likely involved in CM (see Figure 5 and Tables 4 and 5).

**Table 4:** Cerebral malaria (CM)-associated brain inter-endothelial junctions and cell-matrix adhesion molecules known/hypothesized to be matrix metalloproteinase (MMP) substrates

**MMP**	**MMP substrate: junctions**	**Junction type**
MMP-1	Collagen I/II/III/VII/VIII/X; Aggrecan; Entactin; Tenascin	Cell-matrix adhesion
MMP-3	Collagen II/IV/IX/X; Claudin-5; E-cadherin; Elastin; Fibronectin; Laminin; Occludin; Selectin; ZO-1	Cell-matrix adhesion; Adherens junctions; Tight junctions
MMP-8	Collagen I/II/III/V/VII/VIII/X; Claudin-5; Laminin; Occludin; ZO-1	Cell-matrix adhesion; Tight junctions
MMP-9	Collagen IV/V/VII/X/XIV; Aggrecan; Claudin-5; E-cadherin; Elastin; Fibronectin; Laminin; Occludin; Vitronectin; ZO-1	Cell-matrix adhesion; Adherens junctions; Tight junctions
MMP-12	Elastin; Fibronectin; Laminin; Proteoglycans	Cell-matrix adhesion
MMP-13	Collagen I/II/III/IV/V/IX; Aggrecan; Elastin; Fibronectin; Laminin; Tenascin	Cell-matrix adhesion
MMP-14	Collagen I/II/III; E-cadherin; α_v_β_4_ integrin; Aggrecan; Fibronectin; Laminin; Vitronectin	Cell-matrix adhesion; Adherens junctions

**Table 5:** Cerebral malaria (CM)-associated pro-inflammatory molecules known/hypothesized to be matrix metalloproteinase (MMP) substrates

**MMP**	**MMP substrate: cytokine**	**MMP substrate: chemokine**
MMP-1	IL-1β	CCL-2/MCP-1/JE
	TNF-α	CCL-25/TECK
MMP-2	IL-1β	CCL-2/MCP-1/JE
	TGF-β	CCL-11/Eotaxin
	TNF-α	CCL-25/TECK
		CXCL-1/GRO-α/KC
		CXCL-2/GRO-β/MIP-2
		CXCL-12/SDF*-*1
MMP-3	proIL-1β	CCL-2/MCP-1/JE
	proTNF-α	
MMP-7	proTNF-α	
MMP-8		CCL-2/MCP-1/JE
		CXCL-5/ENA-78/LIX
MMP-9	IL-1β	CXCL-1/GRO-α/KC
	IL2-R	CXCL-2/GRO-β/MIP-2
	TGF-β	CXCL-4/PF-4
	proTNF-α	CXCL-5/ENA-78/LIX
		CXCL-10/IP-10
		CXCL-12/SDF*-*1
		CCL-5/RANTES
		CCL-7/MCP-3/MARC
		CCL-17/TARC
		CCL-25/TECK
MMP-10		CCL-25/TECK
MMP-11		CCL-25/TECK
MMP-12		CXCL-3/GRO-γ
		CXCL-9/MIG
		CXCL-10/IP-10
		CXCL-11/I-TAC
MMP-14	proTNF-α	CXCL-8/IL-8
		CXCL-12/SDF*-*1

First, MMPs proteolytically cleave the pro-forms of many cytokines and chemokines reportedly enhanced in CM, including TNF-α, IL-1β, CXCL-8/IL-8. As a result of cleavage, these molecules are shed, activated or functionally potentiated, respectively. Thus, MMPs could contribute to the uncontrolled inflammatory response typical of CM. Consistently, an *in vitro* study using adherent monocytes demonstrated a Hz-dependent pathological auto-enhancing loop established between MMP-9 and TNF-α [180].

Moreover, a growing number of inter-endothelial tight junctions, including occludin, ZO-1, claudin-1 and claudin-5 are known MMP substrates (e.g. MMP-2, MMP-3, MMP-7 and MMP-9), which raises the possibility that MMP activity on these substrates can enhance the permeability of endothelial barriers by destroying these junctions [203-209]. Therefore, enhanced MMP levels in malaria might facilitate BBB leakage. This hypothesis is consistent with data showing that Hz-containing iRBCs reduced human BBB permeability *in vitro*[102], and that Hz and iRBCs enhanced MMP-1, MMP-3 and MMP-9 produced by human microvascular endothelium [194,195].

Finally, MMPs can also influence hemostasis. Both tPA and uPA molecules, responsible for plasminogen conversion to plasmin, as well as tPA/uPA inhibitor PAI-1 can be processed by MMPs [210-212]. As a consequence, MMPs might affect fibrin degradation by plasmin, perhaps explaining thrombotic events that frequently occur during CM.

Interestingly, some molecules such as 4-aminoquinolines and artemisinins, which are currently used for primary therapy of uncomplicated malaria, have displayed MMP-inhibiting properties. Chloroquine treatment reduces MMP-9 serum levels in patients with systemic lupus erythematosus [213]. Artemisinin down-regulates MMP-2 levels in human melanoma cells [214] and MMP-1, MMP-2 and MMP-9 levels in mouse embryonic stem-cells derived from embryoid bodies [215]. Dihydroartemisinin inhibits MMP-2, MMP-9 and MMP-14 expression/activity in human fibrosarcoma cells [216] and MMP-9 expression in human umbilical vein endothelial cells [217]. Finally, artesunate down-regulates MMP-2 and MMP-7 expression in human non-small cell lung cancer [218]. However, it should be noticed that the drug concentrations used in such studies need to be verified and optimized for human clinical trials. This might explain why antimalarial drugs alone cannot prevent CM development.

It is intriguing to explore the idea of targeting MMPs with broad spectrum or specific MMP inhibitors as adjuvant therapy in CM. In the last two decades, a large number of synthetic MMP inhibitors have gone through clinical trials and largely failed as anti-cancer and anti-arthritis drugs due to serious long-term side effects, with only one currently commercially available [219-223]. Hopefully, using combinations of MMP inhibitors with antimalarials could justify lower therapeutic doses of both drugs, thereby reducing their potential side effects whilst still enhancing anti-MMP properties by drug synergy. To date, the effects of MMP inhibitors in CM remain scarce. *In vitro*, the use of a specific synthetic inhibitor of MMP-9 was shown to abrogate Hz-dependent increase of TNF-α in human monocytes, suggesting that MMP-9 inhibition might be useful to counteract pathological inflammation in CM [180]. However, MMP-9 knock-out mice infected with *P. berghei* ANKA did not display any protection from CM development, probably due to the redundant functions of other MMPs which might compensate for the loss of MMP-9 [172]. On the contrary, treatment with broad-spectrum MMP inhibitor BB-94 significantly improved survival of CM mice [172].

Future research aimed at determining the exact role (protective or detrimental) of each MMP during malaria infections will be highly informative. Unfortunately, with the exception of a few cases, specific inhibitors against individual MMPs are currently lacking [224-226]. Some metalloproteinases are also produced by malaria parasites, for example to perform hemoglobin degradation [227]. Therefore, MMP inhibitors may not only influence host but also parasitic pathways. Another issue to be taken in account is represented by the effects of MMPs on other organs than brain. However, it should be noted that the adverse effects of MMP inhibitors documented in other pathologies such as cancer were associated with long-term treatment [220], whereas the time course of drug administration in CM therapy should be reasonably shorter, possibly limiting the development of side effects. A detailed analysis of the role of each protease in physiology and pathology, along with the development of specific inhibitors, could yield novel insights to assess whether specific MMP inhibition might be considered as new adjuvant therapies.

## Conclusion

As suggested by three complementary theories developed over the past century, CM might be a likely consequence of several concomitant phenomena, including iRBC sequestration in brain microvessels, enhanced BBB permeability, and release of pro-inflammatory molecules from host immune cells. Data from *in vitro* and *in vivo* studies suggest that a full BBB breakdown during CM is more likely to occur in mouse than in humans. In the latter case, the BBB appears only mildly impaired as a result of tight junction disruption. MMPs are host proteolytic enzymes involved in degradation of basement membranes, disruption of inter-endothelial tight junctions, and cleavage of a large spectrum of pro-inflammatory, membrane-bound and hemostasis-related molecules, and they may play a crucial role in CM. Further in-depth analysis of the involvement of MMPs in CM might help to design new adjuvant therapies. In this context, MMP inhibitors could prevent BBB leakage and reduce the exacerbated inflammatory response, thus reducing the high mortality rates of CM patients, along with the frequency of neurological sequelae in recovering patients.

## Abbreviations

BBB: Blood–brain barrier (BBB); CNS: Central nervous system; CM: Cerebral malaria; CSF: Cerebral-spinal fluid; ECM: Extracellular matrix; Hz: Hemozoin; HR: Histamine receptor; 15-HETE: 15-hydroxyeicosatetraenoic acid; 4-HNE: 4-hydroxy-2-nonenal; iRBCs: Infected red blood cells; IDO: Indoleamine 2,3-dioxygenase; IFN: Interferon; IL: Interleukin; ICAM-1: Intracellular adhesion molecule-1; MMP: Matrix metalloproteinase; MCP-1: Monocyte chemotactic protein-1; NO: Nitric oxide; NOS2: Nitric oxide synthase type 2; MAPK: Mitogen-activated protein kinase; NF-kB: Nuclear factor-kB; PfEMP-1: *P. falciparum* erythrocyte membrane protein-1; PBCEC: Porcine brain capillary endothelial cell; HBMEC: Human brain microvascular endothelial cell; HUVEC: Human umbilical vascular endothelial cell; ROS: Reactive oxygen species; TIMP: Tissue inhibitor of metalloproteinase; TEER: Trans-endothelial electrical resistance; TGF: Transforming growth factor; TNF: Tumor necrosis factor; VEGF: Vascular endothelial growth factor; uPA: Urokinase-type plasminogen activator; uPAR: Urokinase-type plasminogen activator receptor; VCAM-1: Vascular cellular adhesion molecule-1; ZO-1: Zonula occludens-1.

## Competing interests

The authors declare that they have no competing interests.

## Authors’ information

Manuela Polimeni holds a post-doc fellowship granted by Università di Torino along with Ministero Italiano dell’Università e della Ricerca (MIUR). Mauro Prato holds a professorship granted by Università di Torino along with Azienda Sanitaria Locale-19 (ASL-19) and Compagnia di San Paolo.

## References

[B1] World Health Organization (WHO) World Malaria Report 2013 Geneva: World Health Organization

[B2] Khadjavi A Giribaldi G Prato M From control to eradication of malaria: the end of being stuck in second gear? Asian Pac J Trop Med 2010 3 412 420

[B3] World Health Organization Malaria Policy Advisory Committee and Secretariat Malaria Policy Advisory Committee to the WHO: conclusions and recommendations of September 2012 meeting Malar J 2012 11 424 23253143 10.1186/1475-2875-11-424 PMC3558335

[B4] Mohanty S Patel DK Pati SS Mishra SK Adjuvant therapy in cerebral malaria Indian J Med Res 2006 124 245 260 17085828

[B5] John CC Kutamba E Mugarura K Opoka RO Adjunctive therapy for cerebral malaria and other severe forms of *Plasmodium falciparum* malaria Expert Rev Anti Infect Ther 2010 8 997 1008 20818944 10.1586/eri.10.90 PMC2987235

[B6] Grau GE Craig AG Cerebral malaria pathogenesis: revisiting parasite and host contributions Future Microbiol 2012 7 291 302 22324996 10.2217/fmb.11.155

[B7] Elsheikha HM Khan NA Protozoa traversal of the blood–brain barrier to invade the central nervous system FEMS Microbiol Rev 2010 34 532 553 20337721 10.1111/j.1574-6976.2010.00215.x

[B8] Newton CR Warrel DA Neurological manifestations of falciparum malaria Ann Neurol 1998 43 695 702 9629838 10.1002/ana.410430603

[B9] Gitau EN Newton CR Review article: blood–brain barrier in falciparum malaria Trop Med Int Health 2005 10 285 292 15730513 10.1111/j.1365-3156.2004.01366.x

[B10] World Health Organization (WHO) Guidelines for the treatment of malaria 2006 Geneva: World Health Organization

[B11] Berkley JA Mwangi I Mellington F Mwarumba S Marsh K Cerebral malaria *versus* bacterial meningitis in children with impaired consciousness Q J Med 1999 92 151 157 10.1093/qjmed/92.3.151 10326074

[B12] Newton CR Krishna S Severe falciparum malaria in children: current understanding of pathophysiology and supportive treatment Pharmacol Therap 1998 79 1 53 9719344 10.1016/s0163-7258(98)00008-4

[B13] Coltel N Combes V Hunt NH Grau GE Cerebral malaria - a neurovascular pathology with many riddles still to be solved Curr Neurovasc Res 2004 1 91 110 16185187 10.2174/1567202043480116

[B14] Pino P Taoufiq Z Nitcheu J Vouldoukis I Mazier D Blood–brain barrier breakdown during cerebral malaria: suicide or murder? Thromb Haemost 2005 94 336 340 16113823 10.1160/TH05-05-0354

[B15] Medana IM Turner GD Human cerebral malaria and the blood–brain barrier Int J Parasitol 2006 36 5 10.1016/j.ijpara.2006.02.004 16616145

[B16] Clark IA Budd AC Alleva LM Cowden WB Human malarial disease: a consequence of inflammatory cytokine release Malar J 2006 5 85 17029647 10.1186/1475-2875-5-85 PMC1629020

[B17] van der Heyde HC Nolan J Combes V Gramaglia I Grau GE A unified hypothesis for the genesis of cerebral malaria: sequestration, inflammation and hemostasis leading to microcirculatory dysfunction Trends Parasitol 2006 22 503 508 16979941 10.1016/j.pt.2006.09.002

[B18] Thomson JG Annecke S Observations of the pathology of the central nervous system in malignant tertian malaria, with remarks on certain clinical phenomena J Trop Med Hyg 1926 29 313 346

[B19] MacPherson GG Warrell MJ White NJ Looareesuwan S Warrell DA Human cerebral malaria. A quantitative ultrastructural analysis of parasitized erythrocyte sequestration Am J Pathol 1985 119 385 401 3893148 PMC1888001

[B20] Pongponratn E Riganti M Punpoowong B Aikawa M Microvascular sequestration of parasitized erythrocytes in human falciparum malaria: a pathological study Am J Trop Med Hyg 1991 44 168 175 2012260 10.4269/ajtmh.1991.44.168

[B21] Silamut K Phu NH Whitty C Turner GD Louwrier K Mai NT A quantitative analysis of the microvascular sequestration of malaria parasites in the human brain Am J Pathol 1999 155 395 410 10433933 10.1016/S0002-9440(10)65136-X PMC1866852

[B22] Udeinya IJ Schmidt JA Aikawa M Miller LH Green I Falciparum malaria-infected erythrocytes specifically bind to cultured human endothelial cells Science 1981 213 555 557 7017935 10.1126/science.7017935

[B23] David PH Hommel M Miller LH Udeinya IJ Oligino LD Parasite sequestration in Plasmodium falciparum malaria: spleen and antibody modulation of cytoadherence of infected erythrocytes Proc Natl Acad Sci USA 1983 80 5075 5079 6348780 10.1073/pnas.80.16.5075 PMC384191

[B24] Sherman IW Eda S Winograd E Cytoadherence and sequestration in Plasmodium falciparum: defining the ties that bind Microbes Infect 2003 5 897 909 12919858 10.1016/s1286-4579(03)00162-x

[B25] Chakravorty SJ Craig A The role of ICAM-1 in Plasmodium falciparum cytoadherence Eur J Cell Biol 2005 84 15 27 15724813 10.1016/j.ejcb.2004.09.002

[B26] Dondorp AM Angus BJ Chotivanich K Silamut K Ruangveerayuth R Hardeman MR Red blood cell deformability as a predictor of anemia in severe falciparum malaria Am J Trop Med Hyg 1999 60 733 737 10344643 10.4269/ajtmh.1999.60.733

[B27] Dondorp AM Angus BJ Hardeman MR Chotivanich KT Silamut K Ruangveerayuth R Prognostic significance of reduced red blood cell deformability in severe falciparum malaria Am J Trop Med Hyg 1997 57 507 511 9392587 10.4269/ajtmh.1997.57.507

[B28] Udomsangpetch R Webster HK Pattanapanyasat K Pitchayangkul S Thaithong S Cytoadherence characteristics of rosette-forming Plasmodium falciparum Infect Immun 1992 60 4483 4490 1383150 10.1128/iai.60.11.4483-4490.1992 PMC258192

[B29] Angus BJ Thanikkul K Silamut K White NJ Udomsangpetch R Short report: Rosette formation in Plasmodium ovale infection Am J Trop Med Hyg 1996 55 560 561 8940990 10.4269/ajtmh.1996.55.560

[B30] al-Yaman F Genton B Mokela D Raiko A Kati S Rogerson S Human cerebral malaria: lack of significant association between erythrocyte rosetting and disease severity Trans R Soc Trop Med Hyg 1995 89 55 58 7747308 10.1016/0035-9203(95)90658-4

[B31] Maegraith B Fletcher A The pathogenesis of mammalian malaria Adv Parasitol 1972 10 49 75 4626184 10.1016/s0065-308x(08)60172-4

[B32] Adams S Brown H Turner G Breaking down the blood–brain barrier: signaling a path to cerebral malaria? Trends Parasitol 2002 18 360 366 12377286 10.1016/s1471-4922(02)02353-x

[B33] Enwonwu CO Afolabi BM Salako LA Idigbe EO al-Hassan H Rabiu RA Hyperphenylalaninaemia in children with falciparum malaria QJM 1999 92 495 503 10627868 10.1093/qjmed/92.9.495

[B34] Enwonwu CO Afolabi BM Salako LO Idigbe EO Bashirelah N Increased plasma levels of histidine and histamine in falciparum malaria: relevance to severity of infection J Neural Transm 2000 107 1273 1287 11145003 10.1007/s007020070017

[B35] Beghdadi W Porcherie A Schneider BS Dubayle D Peronet R Huerre M Role of histamine and histamine receptors in the pathogenesis of malaria Med Sci (Paris) 2009 25 377 381 19409190 10.1051/medsci/2009254377

[B36] Beghdadi W Porcherie A Schneider BS Dubayle D Peronet R Huerre M Inhibition of histamine-mediated signaling confers significant protection against severe malaria in mouse models of disease J Exp Med 2008 205 395 408 18227221 10.1084/jem.20071548 PMC2271011

[B37] Beghdadi W Porcherie A Schneider BS Morisset S Dubayle D Peronet R Histamine H(3) receptor-mediated signaling protects mice from cerebral malaria PLoS One 2009 4 6004 10.1371/journal.pone.0006004 PMC2696087 19547708

[B38] Clark IA Virelizier JL Carswell EA Wood PR Possible importance of macrophage-derived mediators in acute malaria Infect Immun 1981 32 1058 1066 6166564 10.1128/iai.32.3.1058-1066.1981 PMC351558

[B39] Clark IA Cowden WB The pathophysiology of falciparum malaria Pharmacol Ther 2003 99 221 260 12888113 10.1016/s0163-7258(03)00060-3

[B40] Clark IA Alleva LM Budd AC Cowden WB Understanding the role of inflammatory cytokines in malaria and related diseases Travel Med Infect Dis 2008 6 67 81 18342278 10.1016/j.tmaid.2007.07.002

[B41] Hansen DS Inflammatory responses associated with the induction of cerebral malaria: lessons from experimental murine models PLoS Pathog 2012 8 e1003045 23300435 10.1371/journal.ppat.1003045 PMC3531491

[B42] Belnoue E Kayibanda M Vigario AM Deschemin JC van Rooijen N Viguier M On the pathogenic role of brain-sequestered alphabeta CD8+ T cells in experimental cerebral malaria J Immunol 2002 169 6369 6375 12444144 10.4049/jimmunol.169.11.6369

[B43] Belnoue E Potter SM Rosa DS Mauduit M Grüner AC Kayibanda M Control of pathogenic CD8+ T cell migration to the brain by IFN-gamma during experimental cerebral malaria Parasite Immunol 2008 30 544 553 18665903 10.1111/j.1365-3024.2008.01053.x

[B44] Claser C Malleret B Gun SY Wong AY Chang ZW Teo P CD8+ T cells and IFN-γ mediate the time-dependent accumulation of infected red blood cells in deep organs during experimental cerebral malaria PLoS One 2011 6 e18720 21494565 10.1371/journal.pone.0018720 PMC3073989

[B45] Hansen DS Bernard NJ Nie CQ Schofield L NK cells stimulate recruitment of CXCR3+ T cells to the brain during Plasmodium berghei-mediated cerebral malaria J Immunol 2007 178 5779 5788 17442962 10.4049/jimmunol.178.9.5779

[B46] Gimenez F Barraud de Lagerie S Fernandez C Pino P Mazier D Tumor necrosis factor alpha in the pathogenesis of cerebral malaria Cell Mol Life Sci 2003 60 1623 1635 14504653 10.1007/s00018-003-2347-x PMC11138823

[B47] Engwerda CR Mynott TL Sawhney S De Souza JB Bickle QD Kaye PM Locally up-regulated lymphotoxin alpha, not systemic tumor necrosis factor alpha, is the principle mediator of murine cerebral malaria J Exp Med 2002 195 1371 1377 12021316 10.1084/jem.20020128 PMC2193758

[B48] Martins YC Carvalho LJ Daniel-Ribeiro CT Challenges in the determination of early predictors of cerebral malaria: lessons from the human disease and the experimental murine models Neuroimmunomodulation 2009 16 134 145 19212133 10.1159/000180268

[B49] Craig AG Grau GE Janse C Kazura JW Milner D Barnwell JW The role of animal models for research on severe malaria PLoS Pathog 2012 8 e1002401 22319438 10.1371/journal.ppat.1002401 PMC3271056

[B50] Suidan GL Pirko I Johnson AJ A potential role for CD8+ T-cells as regulators of CNS vascular permeability Neurol Res 2006 28 250 255 16687049 10.1179/016164106X98116

[B51] Suidan GL Mcdole JR Chen Y Pirko I Johnson AJ Induction of blood brain barrier tight junction protein alterations by CD8 T cells PLoS One 2008 3 e3037 18725947 10.1371/journal.pone.0003037 PMC2516328

[B52] Suidan GL Dickerson JW Chen Y McDole JR Tripathi P Pirko I CD8 T cell-initiated vascular endothelial growth factor expression promotes central nervous system vascular permeability under neuroinflammatory conditions J Immunol 2010 184 1031 1040 20008293 10.4049/jimmunol.0902773 PMC2896014

[B53] Lundie RJ de Koning-Ward TF Davey GM Nie CQ Hansen DS Lau LS Blood-stage Plasmodium infection induces CD8+ T lymphocytes to parasite-expressed antigens, largely regulated by CD8alpha + dendritic cells Proc Natl Acad Sci USA 2008 105 14509 14514 18799734 10.1073/pnas.0806727105 PMC2567226

[B54] Miyakoda M Kimura D Yuda M Chinzei Y Shibata Y Honma K Yui K Malaria-specific and nonspecific activation of CD8+ T cells during blood stage of Plasmodium berghei infection J Immunol 2008 181 1420 1428 18606696 10.4049/jimmunol.181.2.1420

[B55] Howland SW Poh CM Gun SY Claser C Malleret B Shastri N Brain microvessel cross-presentation is a hallmark of experimental cerebral malaria EMBO Mol Med 2013 5 916 931 23681698 10.1002/emmm.201202273 PMC3721469

[B56] Jambou R Combes V Jambou MJ Weksler BB Couraud PO Grau GE *Plasmodium falciparum* adhesion on human brain microvascular endothelial cells involves transmigration-like cup formation and induces opening of intercellular junctions PLoS Pathog 2010 6 e1001021 20686652 10.1371/journal.ppat.1001021 PMC2912387

[B57] Yui K Cross-presentation of malaria antigen by brain microvessels: why CD8(+) T cells are critical for the pathogenesis of experimental cerebral malaria EMBO Mol Med 2013 5 899 901 23740752 10.1002/emmm.201302849 PMC3721466

[B58] Grau GE Fajardo LF Piguet PF Allet B Lambert PH Vassalli P Tumor necrosis factor (cachectin) as an essential mediator in murine cerebral malaria Science 1987 237 1210 1212 3306918 10.1126/science.3306918

[B59] Rudin W Eugster HP Bordmann G Bonato J Müller M Yamage M Ryffel B Resistance to cerebral malaria in tumor necrosis factor-alpha/beta-deficient mice is associated with a reduction of intercellular adhesion molecule-1 up-regulation and T helper type 1 response Am J Pathol 1997 150 257 266 9006341 PMC1858518

[B60] de Miranda AS Lacerda-Queiroz N de Carvalho Vilela M Rodrigues DH Rachid MA Quevedo J Teixeira AL Anxiety-like behavior and proinflammatory cytokine levels in the brain of C57BL/6 mice infected with Plasmodium berghei (strain ANKA) Neurosci Lett 2011 491 202 206 21256928 10.1016/j.neulet.2011.01.038

[B61] Wu JJ Chen G Liu J Wang T Zheng W Cao YM Natural regulatory T cells mediate the development of cerebral malaria by modifying the pro-inflammatory response Parasitol Int 2010 59 232 241 20219695 10.1016/j.parint.2010.02.007

[B62] Parekh SB Bubb WA Hunt NH Rae C Brain metabolic markers reflect susceptibility status in cytokine gene knockout mice with murine cerebral malaria Int J Parasitol 2006 36 1409 1418 16934816 10.1016/j.ijpara.2006.07.004

[B63] Amante FH Haque A Stanley AC Rivera Fde L Randall LM Wilson YA Immune-mediated mechanisms of parasite tissue sequestration during experimental cerebral malaria J Immunol 2010 185 3632 3642 20720206 10.4049/jimmunol.1000944

[B64] Esamai F Ernerudh J Janols H Welin S Ekerfelt C Mining S Forsberg P Cerebral malaria in children: serum and cerebrospinal fluid TNF-alpha and TGF-beta levels and their relationship to clinical outcome J Trop Pediatr 2003 49 216 223 12929882 10.1093/tropej/49.4.216

[B65] Armah HB Wilson NO Sarfo BY Powell MD Bond VC Anderson W Cerebrospinal fluid and serum biomarkers of cerebral malaria mortality in Ghanaian children Malar J 2007 6 147 17997848 10.1186/1475-2875-6-147 PMC2186349

[B66] Jain V Armah HB Tongren JE Ned RM Wilson NO Crawford S Plasma IP-10, apoptotic and angiogenic factors associated with fatal cerebral malaria in India Malar J 2008 7 83 18489763 10.1186/1475-2875-7-83 PMC2405803

[B67] Thuma PE van Dijk J Bucala R Debebe Z Nekhai S Kuddo T Distinct clinical and immunologic profiles in severe malarial anemia and cerebral malaria in Zambia J Infect Dis 2011 203 211 219 21288821 10.1093/infdis/jiq041 PMC3071068

[B68] Lovegrove FE Tangpukdee N Opoka RO Lafferty EI Rajwans N Hawkes M Serum angiopoietin-1 and -2 levels discriminate cerebral malaria from uncomplicated malaria and predict clinical outcome in African children PLoS One 2009 4 e4912 19300530 10.1371/journal.pone.0004912 PMC2657207

[B69] Prakash D Fesel C Jain R Cazenave PA Mishra GC Pied S Clusters of cytokines determine malaria severity in Plasmodium falciparum-infected patients from endemic areas of Central India J Infect Dis 2006 194 198 207 16779726 10.1086/504720

[B70] Stevenson MM Su Z Sam H Mohan K Modulation of host responses to blood-stage malaria by interleukin-12: from therapy to adjuvant activity Microbes Infect 2001 3 49 59 11226854 10.1016/s1286-4579(00)01354-x

[B71] Marquet S Doumbo O Cabantous S Poudiougou B Argiro L Safeukui I A functional promoter variant in IL12B predisposes to cerebral malaria Hum Mol Genet 2008 17 2190 2195 18413324 10.1093/hmg/ddn118

[B72] Sanni LA The role of cerebral oedema in the pathogenesis of cerebral malaria Redox Rep 2001 6 137 142 11523587 10.1179/135100001101536238

[B73] Hansen AM Driussi C Turner V Takikawa O Hunt NH Tissue distribution of indoleamine 2,3-dioxygenase in normal and malaria-infected tissue Redox Rep 2000 5 112 115 10939286 10.1179/135100000101535384

[B74] Anstey NM Weinberg JB Hassanali MY Mwaikambo ED Manyenga D Misukonis MA Nitric oxide in Tanzanian children with malaria: inverse relationship between malaria severity and nitric oxide production/nitric oxide synthase type 2 expression J Exp Med 1996 184 557 567 8760809 10.1084/jem.184.2.557 PMC2192721

[B75] Hobbs MR Udhayakumar V Levesque MC Booth J Roberts JM Tkachuk AN A new NOS2 promoter polymorphism associated with increased nitric oxide production and protection from severe malaria in Tanzanian and Kenyan children Lancet 2002 360 1468 1475 12433515 10.1016/S0140-6736(02)11474-7

[B76] Lopansri BK Anstey NM Weinberg JB Stoddard GJ Hobbs MR Levesque MC Low plasma arginine concentrations in children with cerebral malaria and decreased nitric oxide production Lancet 2003 361 676 678 12606182 10.1016/S0140-6736(03)12564-0

[B77] Yeo TW Lampah DA Gitawati R Tjitra E Kenangalem E McNeil YR Impaired nitric oxide bioavailability and L-arginine reversible endothelial dysfunction in adults with falciparum malaria J Exp Med 2007 204 2693 2704 17954570 10.1084/jem.20070819 PMC2118490

[B78] Sobolewski P Gramaglia I Frangos J Intaglietta M van der Heyde HC Nitric oxide bioavailability in malaria Trends Parasitol 2005 21 415 422 16039159 10.1016/j.pt.2005.07.002

[B79] Gramaglia I Sobolewski P Meays D Contreras R Nolan JP Frangos JA Low nitric oxide bioavailability contributes to the genesis of experimental cerebral malaria Nat Med 2006 12 1417 1422 17099710 10.1038/nm1499

[B80] Serghides L Kim H Lu Z Kain DC Miller C Francis RC Inhaled nitric oxide reduces endothelial activation and parasite accumulation in the brain, and enhances survival in experimental cerebral malaria PLoS One 2011 6 e27714 22110737 10.1371/journal.pone.0027714 PMC3218025

[B81] Zanini GM Cabrales P Barkho W Frangos JA Carvalho LJ Exogenous nitric oxide decreases brain vascular inflammation, leakage and venular resistance during Plasmodium berghei ANKA infection in mice J Neuroinflamm 2011 8 66 10.1186/1742-2094-8-66 PMC3118350 21649904

[B82] Yeo TW Lampah DA Rooslamiati I Gitawati R Tjitra E Kenangalem E A randomized pilot study of L-arginine infusion in severe falciparum malaria: preliminary safety, efficacy and pharmacokinetics PLoS One 2013 8 e69587 23922746 10.1371/journal.pone.0069587 PMC3726665

[B83] Das BS Mohanty S Mishra SK Patnaik JK Satpathy SK Mohanty D Bose TK Increased cerebrospinal fluid protein and lipid peroxidation products in patients with cerebral malaria Trans R Soc Trop Med Hyg 1991 85 733 734 1801339 10.1016/0035-9203(91)90436-3

[B84] Das BS Patnaik JK Mohanty S Mishra SK Mohanty D Satpathy SK Plasma antioxidants and lipid peroxidation products in falciparum malaria Am J Trop Med Hyg 1993 49 720 725 8279640 10.4269/ajtmh.1993.49.720

[B85] Mishra NC Kabilan L Sharma A Oxidative stress and malaria-infected erythrocytes Indian J Malariol 1994 31 77 87 7713262

[B86] Postma NS Mommers EC Eling WM Zuidema J Oxidative stress in malaria; implications for prevention and therapy Pharm World Sci 1996 18 121 129 8873227 10.1007/BF00717727

[B87] Thumwood CM Hunt NH Cowden WB Clark IA Antioxidants can prevent cerebral malaria in Plasmodium berghei-infected mice Br J Exp Pathol 1989 70 293 303 2669924 PMC2040571

[B88] Reis PA Comim CM Hermani F Silva B Barichello T Portella AC Cognitive dysfunction is sustained after rescue therapy in experimental cerebral malaria, and is reduced by additive antioxidant therapy PLoS Pathog 2010 6 e1000963 20585569 10.1371/journal.ppat.1000963 PMC2891838

[B89] Sanni LA Fu S Dean RT Bloomfield G Stocker R Chaudhri G Are reactive oxygen species involved in the pathogenesis of murine cerebral malaria? J Infect Dis 1999 179 217 222 9841842 10.1086/314552

[B90] Potter SM Mitchell AJ Cowden WB Sanni LA Dinauer M de Haan JB Phagocyte-derived reactive oxygen species do not influence the progression of murine blood-stage malaria infections Infect Immun 2005 73 4941 4947 16041008 10.1128/IAI.73.8.4941-4947.2005 PMC1201219

[B91] Linares M Marín-García P Martínez-Chacón G Pérez-Benavente S Puyet A Diez A Bautista JM Glutathione peroxidase contributes with heme oxygenase-1 to redox balance in mouse brain during the course of cerebral malaria Biochim Biophys Acta 1832 2013 2009 2018 10.1016/j.bbadis.2013.07.010 23872112

[B92] Taoufiq Z Pino P Dugas N Conti M Tefit M Mazier D Vouldoukis I Transient supplementation of superoxide dismutase protects endothelial cells against Plasmodium falciparum-induced oxidative stress Mol Biochem Parasitol 2006 150 166 173 16930739 10.1016/j.molbiopara.2006.07.008

[B93] Tripathi AK Sullivan DJ Stins MF Plasmodium falciparum-infected erythrocytes increase intercellular adhesion molecule 1 expression on brain endothelium through NF-kappaB Infect Immun 2006 74 3262 3270 16714553 10.1128/IAI.01625-05 PMC1479273

[B94] Narsaria N Mohanty C Das BK Mishra SP Prasad R Oxidative stress in children with severe malaria J Trop Pediatr 2012 58 147 150 21602230 10.1093/tropej/fmr043

[B95] Abbott NJ Friedman A Overview and introduction: the blood–brain barrier in health and disease Epilepsia 2012 53 Suppl 6 1 6 23134489 10.1111/j.1528-1167.2012.03696.x PMC3625728

[B96] Luissint AC Artus C Glacial F Ganeshamoorthy K Couraud PO Tight junctions at the blood brain barrier: physiological architecture and disease-associated dysregulation Fluids Barriers CNS 2012 9 23 23140302 10.1186/2045-8118-9-23 PMC3542074

[B97] Cucullo L Aumayr B Rapp E Janigro D Drug delivery and *in vitro* models of the blood–brain barrier Curr Opin Drug Discov Devel 2005 8 89 99 15679176

[B98] Renia L Howland SW Claser C Gruner AC Suwanarusk R Hui Teo T Russell B Ng LF Cerebral malaria – Mysteries at the blood-brain barrier Virulence 2012 3 193 201 22460644 10.4161/viru.19013 PMC3396698

[B99] Treeratanapiboon L Psathaki K Wegener J Looareesuwan S Galla HJ Udomsangpetch R *In vitro* study of malaria parasite induced disruption of blood–brain barrier Biochem Biophys Res Commun 2005 335 810 818 16105659 10.1016/j.bbrc.2005.07.151

[B100] Susomboon P Maneerat Y Dekumyoy P Kalambaheti T Iwagami M Komaki-Yasuda K Down-regulation of tight junction mRNAs in human endothelial cells co-cultured with Plasmodium falciparum-infected erythrocytes Parasitol Int 2006 55 107 112 16388977 10.1016/j.parint.2005.11.054

[B101] Wassmer SC Combes V Candal FJ Juhan-Vague I Grau GE Platelets potentiate brain endothelial alterations induced by Plasmodium falciparum Infect Immun 2006 74 645 653 16369021 10.1128/IAI.74.1.645-653.2006 PMC1346683

[B102] Tripathi AK Sullivan DJ Stins MF Plasmodium falciparum-infected erythrocytes decrease the integrity of human blood–brain barrier endothelial cell monolayers J Infect Dis 2007 195 942 950 17330783 10.1086/512083

[B103] Gillrie MR Krishnegowda G Lee K Buret AG Robbins SM Looareesuwan S Src-family kinase dependent disruption of endothelial barrier function by Plasmodium falciparum merozoite proteins Blood 2007 110 3426 3435 17693580 10.1182/blood-2007-04-084582 PMC2200906

[B104] Tripathi AK Sha W Shulaev V Stins MF Sullivan DJ Jr Plasmodium falciparum-infected erythrocytes induce NF-kappaB regulated inflammatory pathways in human cerebral endothelium Blood 2009 114 4243 4252 19713460 10.1182/blood-2009-06-226415 PMC2925626

[B105] Zougbédé S Miller F Ravassard P Rebollo A Cicéron L Couraud PO Metabolic acidosis induced by Plasmodium falciparum intraerythrocytic stages alters blood–brain barrier integrity J Cereb Blood Flow Metab 2011 31 514 526 20683453 10.1038/jcbfm.2010.121 PMC3049507

[B106] Wassmer SC Moxon CA Taylor T Grau GE Molyneux ME Craig AG Vascular endothelial cells cultured from patients with cerebral or uncomplicated malaria exhibit differential reactivity to TNF Cell Microbiol 2011 13 198 209 21029292 10.1111/j.1462-5822.2010.01528.x PMC3041929

[B107] Claessens A Rowe JA Selection of Plasmodium falciparum parasites for cytoadhesion to human brain endothelial cells J Vis Exp 2012 59 e3122 22230803 10.3791/3122 PMC3369769

[B108] El-Assaad F Wheway J Mitchell AJ Lou J Hunt NH Combes V Grau GE Cytoadherence of Plasmodium berghei-infected red blood cells to murine brain and lung microvascular endothelial cells *in vitro* Infect Immun 2013 81 3984 3991 23940206 10.1128/IAI.00428-13 PMC3811819

[B109] Migasena P Maegraith BG The movement of radioactive albumin (I131 R.H.S.A.) from blood into C.S.F. and vice versa by dilution method in normal and Plasmodium knowlesi infected Macaca mulatta Med J Malaya 1968 22 250 4234388

[B110] Migasena P Maegraith BG Factor affecting on the movement of protein across the blood: brain: C.S.F. barriers in Plasmodium knowlesi infected Macaca mulatta Med J Malaya 1968 22 251 4234389

[B111] Migasena P Maegraith BG The movement of fluorescent isothiocyanate (F.I.T.C.) labelled human albumin from blood into brain tissue examined by fluorescent technique in normal and Plasmodium knowlesi infected Macaca mulatta Med J Malaya 1968 22 251 4234390

[B112] Fujioka H Millet P Maeno Y Nakazawa S Ito Y Howard RJ A nonhuman primate model for human cerebral malaria: rhesus monkeys experimentally infected with Plasmodium fragile Exp Parasitol 1994 78 371 376 7515825 10.1006/expr.1994.1040

[B113] Moreno A Cabrera-Mora M Garcia A Orkin J Strobert E Barnwell JW Plasmodium coatneyi in rhesus macaques replicates the multisystemic dysfunction of severe malaria in humans Infect Immun 2013 81 1889 1904 23509137 10.1128/IAI.00027-13 PMC3676004

[B114] Tongren JE Yang C Collins WE Sullivan JS Lal AA Xiao L Expression of proinflammatory cytokines in four regions of the brain in Macaque mulatta (rhesus) monkeys infected with Plasmodium coatneyi Am J Trop Med Hyg 2000 62 530 534 11220773 10.4269/ajtmh.2000.62.530

[B115] Thumwood CM Hunt NH Clark IA Cowden WB Breakdown of the blood–brain barrier in murine cerebral malaria Parasitology 1988 96 579 589 2457201 10.1017/s0031182000080203

[B116] Polder T Jerusalem C Eling W Topographical distribution of the cerebral lesions in mice infected with Plasmodium berghei Tropenmed Parasitol 1983 34 235 243 6364514

[B117] Polder TW Eling WM Kubat K Jerusalem CR Histochemistry of cerebral lesions in mice infected with Plasmodium berghei Trop Med Parasitol 1988 39 277 283 3067319

[B118] Polder TW Eling WM Curfs JH Jerusalem CR Wijers-Rouw M Ultrastructural changes in the blood–brain barrier of mice infected with Plasmodium berghei Acta Leiden 1992 60 31 46 1485495

[B119] Neill AL Hunt NH Pathology of fatal and resolving Plasmodium berghei cerebral malaria in mice Parasitology 1992 105 165 175 1280805 10.1017/s0031182000074072

[B120] Penet MF Viola A Confort-Gouny S Le Fur Y Duhamel G Kober F Imaging experimental cerebral malaria *in vivo*: significant role of ischemic brain oedema J Neurosci 2005 25 7352 7358 16093385 10.1523/JNEUROSCI.1002-05.2005 PMC6725296

[B121] Schmidt KE Schumak B Specht S Dubben B Limmer A Hoerauf A Induction of pro-inflammatory mediators in Plasmodium berghei infected BALB/c mice breaks blood–brain-barrier and leads to cerebral malaria in an IL-12 dependent manner Microbes Infect 2011 13 828 836 21609776 10.1016/j.micinf.2011.04.006

[B122] Lacerda-Queiroz N Lima OC Carneiro CM Vilela MC Teixeira AL Teixeira-Carvalho A Plasmodium berghei NK65 induces cerebral leukocyte recruitment *in vivo*: an intravital microscopic study Acta Trop 2011 120 31 39 21722620 10.1016/j.actatropica.2011.04.020

[B123] Keswani T Bhattacharyya A Splenocyte apoptosis in Plasmodium berghei ANKA infection: possible role of TNF-α and TGF-β Parasite Immunol 2013 35 73 90 23009201 10.1111/pim.12005

[B124] Nacer A Movila A Baer K Mikolajczak SA Kappe SH Frevert U Neuroimmunological blood brain barrier opening in experimental cerebral malaria PLoS Pathog 2012 8 e1002982 23133375 10.1371/journal.ppat.1002982 PMC3486917

[B125] Owens T Bechmann I Engelhardt B Perivascular spaces and the two steps to neuroinflammation J Neuropathol Exp Neurol 2008 67 1113 1121 19018243 10.1097/NEN.0b013e31818f9ca8

[B126] Warrell DA Looareesuwan S Phillips RE White NJ Warrell MJ Chapel HM Function of the blood-cerebrospinal fluid barrier in human cerebral malaria: rejection of the permeability hypothesis Am J Trop Med Hyg 1986 35 882 889 2429567 10.4269/ajtmh.1986.35.882

[B127] Brown HC Chau TT Mai NT Day NP Sinh DX White NJ Blood–brain barrier function in cerebral malaria and CNS infections in Vietnam Neurology 2000 55 104 111 10891914 10.1212/wnl.55.1.104

[B128] Badibanga B Dayal R Depierreux M Pidival G Lambert PH Principle immunological factors and the blood–brain barrier in cerebral malaria in children in endemic countries (Zaïre) Ann Soc Belg Med Trop 1986 66 23 37 3521502

[B129] Brown H Rogerson S Taylor T Tembo M Mwenechanya J Molyneux M Blood–brain barrier function in cerebral malaria in Malawian children Am J Trop Med Hyg 2001 64 207 213 11442219 10.4269/ajtmh.2001.64.207

[B130] Mturi N Keir G Maclennan CA Ross A Willis AC Elford BC Cerebrospinal Fluid Studies in Kenyan Children with Severe Falciparum Malaria Open Trop Med J 2008 1 56 62 20396606 10.2174/1874315300801010056 PMC2854806

[B131] Dorovini-Zis K Schmidt K Huynh H Fu W Whitten RO Milner D The neuropathology of fatal cerebral malaria in Malawian children Am J Pathol 2011 178 2146 2158 21514429 10.1016/j.ajpath.2011.01.016 PMC3081150

[B132] Walker O Salako LA Sowunmi A Thomas JO Sodeine O Bondi FS Prognostic risk factors and post mortem findings in cerebral malaria in children Trans R Soc Trop Med Hyg 1992 86 491 493 1475813 10.1016/0035-9203(92)90082-n

[B133] Pongponratn E Turner GD Day NP Phu NH Simpson JA Stepniewska K An ultrastructural study of the brain in fatal Plasmodium falciparum malaria Am J Trop Med Hyg 2003 69 345 359 14640492

[B134] Medana IM Day NP Sachanonta N Mai NT Dondorp AM Pongponratn E Coma in fatal adult human malaria is not caused by cerebral oedema Malar J 2011 10 267 21923924 10.1186/1475-2875-10-267 PMC3182981

[B135] Brown H Hien TT Day N Mai NT Chuong LV Chau TT Evidence of blood–brain barrier dysfunction in human cerebral malaria Neuropathol Appl Neurobiol 1999 25 331 340 10476050 10.1046/j.1365-2990.1999.00188.x

[B136] Newton CR Peshu N Kendall B Kirkham FJ Sowunmi A Waruiru C Brain swelling and ischaemia in Kenyans with cerebral malaria Arch Dis Child 1994 70 281 287 8185359 10.1136/adc.70.4.281 PMC1029778

[B137] Cordoliani YS Sarrazin JL Felten D Caumes E Leveque C Fisch A MR of cerebral malaria AJNR Am J Neuroradiol 1998 19 871 874 9613502 PMC8337584

[B138] Ngoungou EB Dulac O Poudiougou B Druet-Cabanac M Dicko A Mamadou Traore A Epilepsy as a consequence of cerebral malaria in area in which malaria is endemic in Mali, West Africa Epilepsia 2006 47 873 879 16686652 10.1111/j.1528-1167.2006.00558.x

[B139] Lacout A Guidoux C Carlier RY Posterior reversible encephalopathy syndrome in neuro-malaria Indian J Radiol Imaging 2010 20 198 201 21042444 10.4103/0971-3026.69357 PMC2963746

[B140] Potchen MJ Birbeck GL Demarco JK Kampondeni SD Beare N Molyneux ME Taylor TE Neuroimaging findings in children with retinopathy-confirmed cerebral malaria Eur J Radiol 2010 74 262 268 19345538 10.1016/j.ejrad.2009.02.010 PMC3786197

[B141] Rasalkar DD Paunipagar BK Sanghvi D Sonawane BD Loniker P Magnetic resonance imaging in cerebral malaria: a report of four cases Br J Radiol 2011 84 380 385 21415303 10.1259/bjr/85759874 PMC3473476

[B142] Potchen MJ Kampondeni SD Seydel KB Birbeck GL Hammond CA Bradley WG Acute brain MRI findings in 120 Malawian children with cerebral malaria: new insights into an ancient disease AJNR Am J Neuroradiol 2012 33 1740 1746 22517285 10.3174/ajnr.A3035 PMC3779545

[B143] Kampondeni SD Potchen MJ Beare NA Seydel KB Glover SJ Taylor TE Birbeck GL MRI findings in a cohort of brain injured survivors of pediatric cerebral malaria Am J Trop Med Hyg 2013 88 542 546 23339204 10.4269/ajtmh.12-0538 PMC3592538

[B144] Frevert U Nacer A Cabrera M Movila A Leberl M Imaging Plasmodium immunobiology in the liver, brain, and lung Parasitol Int 2014 63 171 186 24076429 10.1016/j.parint.2013.09.013 PMC3876283

[B145] Taylor TE Fu WJ Carr RA Whitten RO Mueller JS Fosiko NG Differentiating the pathologies of cerebral malaria by postmortem parasite counts Nat Med 2004 10 143 145 14745442 10.1038/nm986

[B146] Riley EM Couper KN Helmby H Hafalla JC de Souza JB Langhorne J Neuropathogenesis of human and murine malaria Trends Parasitol 2010 26 277 278 20338809 10.1016/j.pt.2010.03.002

[B147] Cabrales P Zanini GM Meays D Frangos JA Carvalho LJ Murine cerebral malaria is associated with a vasospasm-like microcirculatory dysfunction, and survival upon rescue treatment is markedly increased by nimodipine Am J Pathol 2010 176 1306 1315 20110412 10.2353/ajpath.2010.090691 PMC2832151

[B148] Hawkes M Elphinstone RE Conroy AL Kain KC Contrasting pediatric and adult cerebral malaria: the role of the endothelial barrier Virulence 2013 4 543 555 23924893 10.4161/viru.25949 PMC5359751

[B149] Saunders NR Habgood MD Dziegielewska KM Barrier mechanisms in the brain, II. Immature brain Clin Exp Pharmacol Physiol 1999 26 85 91 10065326 10.1046/j.1440-1681.1999.02987.x

[B150] Ek CJ Dziegielewska KM Habgood MD Saunders NR Barriers in the developing brain and Neurotoxicology Neurotoxicology 2012 33 586 604 22198708 10.1016/j.neuro.2011.12.009

[B151] Szklarczyk A Stins M Milward EA Ryu H Fitzsimmons C Sullivan D Glial activation and matrix metalloproteinase release in cerebral malaria J Neurovirol 2007 13 2 10 17454443 10.1080/13550280701258084

[B152] Prato M Giribaldi G Matrix metalloproteinase-9 and haemozoin: wedding rings for human host and Plasmodium falciparum parasite in complicated malaria J Trop Med 2011 2011 628435 21760809 10.1155/2011/628435 PMC3134216

[B153] Geurts N Opdenakker G Van den Steen PE Matrix metalloproteinases as therapeutic targets in protozoan parasitic infections Pharmacol Ther 2012 133 257 279 22138604 10.1016/j.pharmthera.2011.11.008

[B154] Piña-Vázquez C Reyes-López M Ortíz-Estrada G de la Garza M Serrano-Luna J Host-parasite interaction: parasite-derived and -induced proteases that degrade human extracellular matrix J Parasitol Res 2012 2012 748206 22792442 10.1155/2012/748206 PMC3390111

[B155] Nagase H Woessner JF Jr Matrix metalloproteinases J Biol Chem 1999 274 21491 21494 10419448 10.1074/jbc.274.31.21491

[B156] Rosenberg GA Matrix metalloproteinases in neuroinflammation Glia 2002 39 279 291 12203394 10.1002/glia.10108

[B157] Woessner JF Jr MMPs and TIMPs–an historical perspective Mol Biotechnol 2002 22 33 49 12353913 10.1385/MB:22:1:033

[B158] Rosenberg GA Yang Y Vasogenic oedema due to tight junction disruption by matrix metalloproteinases in cerebral ischemia Neurosurg Focus 2007 22 E4 17613235 10.3171/foc.2007.22.5.5

[B159] Klein T Bischoff R Physiology and pathophysiology of matrix metalloproteases Amino Acids 2011 41 271 290 20640864 10.1007/s00726-010-0689-x PMC3102199

[B160] Peng WJ Yan JW Wan YN Wang BX Tao JH Yang GJ Matrix metalloproteinases: a review of their structure and role in systemic sclerosis J Clin Immunol 2012 32 1409 1414 22767184 10.1007/s10875-012-9735-7

[B161] Mannello F Medda V Nuclear localization of matrix metalloproteinases Prog Histochem Cytochem 2012 47 27 58 22226510 10.1016/j.proghi.2011.12.002

[B162] Marco M Fortin C Fulop T Membrane-type matrix metalloproteinases: key mediators of leukocyte function J Leukoc Biol 2013 94 237 246 23695309 10.1189/jlb.0612267

[B163] Siefert SA Sarkar R Matrix metalloproteinases in vascular physiology and disease Vascular 2012 20 210 216 22896663 10.1258/vasc.2011.201202

[B164] Van Wart HE Birkedal-Hansen H The cysteine switch: a principle of regulation of metalloproteinase activity with potential applicability to the entire matrix metalloproteinase gene family Proc Natl Acad Sci USA 1990 87 5578 5582 2164689 10.1073/pnas.87.14.5578 PMC54368

[B165] Van den Steen PE Dubois B Nelissen I Rudd PM Dwek RA Opdenakker G Biochemistry and molecular biology of gelatinase B or matrix metalloproteinase-9 (MMP-9) Crit Rev Biochem Mol Biol 2002 37 375 536 12540195 10.1080/10409230290771546

[B166] Nagase H Cell surface activation of progelatinase A (proMMP-2) and cell migration Cell Res 1998 8 179 186 9791731 10.1038/cr.1998.18

[B167] Nagase H Visse R Murphy G Structure and function of matrix metalloproteinases and TIMPs Cardiovasc Res 2006 69 562 573 16405877 10.1016/j.cardiores.2005.12.002

[B168] Zhao H Bernardo MM Osenkowski P Sohail A Pei D Nagase H Differential inhibition of membrane type 3 (MT3)-matrix metalloproteinase (MMP) and MT1-MMP by tissue inhibitor of metalloproteinase (TIMP)-2 and TIMP-3 rgulates pro-MMP-2 activation J Biol Chem 2004 279 8592 8601 14681236 10.1074/jbc.M308708200

[B169] Cauwe B Van den Steen PE Opdenakker G The biochemical, biological, and pathological kaleidoscope of cell surface substrates processed by matrix metalloproteinases Crit Rev Biochem Mol Biol 2007 42 113 185 17562450 10.1080/10409230701340019

[B170] Van Lint P Libert C Chemokine and cytokine processing by matrix metalloproteinases and its effect on leukocyte migration and inflammation J Leukoc Biol 2007 82 1375 1381 17709402 10.1189/jlb.0607338

[B171] Butler GS Overall CM Updated biological roles for matrix metalloproteinases and new “intracellular” substrates revealed by degradomics Biochemistry 2009 48 10830 10845 19817485 10.1021/bi901656f

[B172] Van den Steen PE Van Aelst I Starckx S Maskos K Opdenakker G Pagenstecher A Matrix metalloproteinases, tissue inhibitors of MMPs and TACE in experimental cerebral malaria Lab Invest 2006 86 873 888 16865090 10.1038/labinvest.3700454

[B173] Deroost K Tyberghein A Lays N Noppen S Schwarzer E Vanstreels E Hemozoin Induces Lung Inflammation and Correlates with Malaria-Associated Acute Respiratory Distress Syndrome Am J Respir Cell Mol Biol 2013 48 589 600 23328641 10.1165/rcmb.2012-0450OC

[B174] Piguet PF Da Laperrousaz C Vesin C Tacchini-Cottier F Senaldi G Grau GE Delayed mortality and attenuated thrombocytopenia associated with severe malaria in urokinase- and urokinase receptor-deficient mice Infect Immun 2000 68 3822 3829 10858190 10.1128/iai.68.7.3822-3829.2000 PMC101654

[B175] Egan TJ Recent advances in understanding the mechanism of hemozoin (malaria pigment) formation J Inorg Biochem 2008 102 1288 1299 18226838 10.1016/j.jinorgbio.2007.12.004

[B176] Sullivan AD Ittarat I Meshnick SR Patterns of haemozoin accumulation in tissue Parasitology 1996 112 Pt 3 285 294 8728992 10.1017/s003118200006580x

[B177] Carney CK Schrimpe AC Halfpenny K Harry RS Miller CM Broncel M The basis of the immunomodulatory activity of malaria pigment (hemozoin) J Biol Inorg Chem 2006 11 917 929 16868743 10.1007/s00775-006-0147-0

[B178] Schrimpe AC Wright DW Comparative analysis of gene expression changes mediated by individual constituents of hemozoin Chem Res Toxicol 2009 22 433 445 19191707 10.1021/tx8002752 PMC4208677

[B179] Schrimpe AC Wright DW Differential gene expression mediated by 15-hydroxyeicosatetraenoic acid in LPS-stimulated RAW 264.7 cells Malar J 2009 8 195 19671186 10.1186/1475-2875-8-195 PMC2743705

[B180] Prato M Giribaldi G Polimeni M Gallo V Arese P Phagocytosis of hemozoin enhances matrix metalloproteinase-9 activity and TNF-alpha production in human monocytes: role of matrix metalloproteinases in the pathogenesis of falciparum malaria J Immunol 2005 175 6436 6442 16272296 10.4049/jimmunol.175.10.6436

[B181] Prato M Gallo V Giribaldi G Arese P Phagocytosis of haemozoin (malarial pigment) enhances metalloproteinase-9 activity in human adherent monocytes: role of IL-1beta and 15-HETE Malar J 2008 7 157 18710562 10.1186/1475-2875-7-157 PMC2529304

[B182] Prato M Gallo V Giribaldi G Aldieri E Arese P Role of the NF-κB transcription pathway in the haemozoin- and 15-HETE-mediated activation of matrix metalloproteinase-9 in human adherent monocytes Cell Microbiol 2010 12 1780 1791 20678173 10.1111/j.1462-5822.2010.01508.x

[B183] Giribaldi G Prato M Ulliers D Gallo V Schwarzer E Akide-Ndunge OB Involvement of inflammatory chemokines in survival of human monocytes fed with malarial pigment Infect Immun 2010 78 4912 4921 20732999 10.1128/IAI.00455-10 PMC2976350

[B184] Giribaldi G Valente E Khadjavi A Polimeni M Prato M Macrophage inflammatory protein-1alpha mediates matrix metalloproteinase-9 enhancement in human adherent monocytes fed with malarial pigment Asian Pac J Trop Med 2011 4 925 930 22118025 10.1016/S1995-7645(11)60220-4

[B185] Khadjavi A Valente E Giribaldi G Prato M Involvement of p38 MAPK in natural haemozoin- and 15-HETE-dependent MMP-9 enhancement in human adherent monocytes Cell Biochem Funct 2014 32 5 15 23468369 10.1002/cbf.2963

[B186] Polimeni M Valente E Ulliers D Opdenakker G Van den Steen PE Giribaldi G Prato M Natural Haemozoin Induces Expression and Release of Human Monocyte Tissue Inhibitor of Metalloproteinase-1 PLoS ONE 2013 8 e71468 23967215 10.1371/journal.pone.0071468 PMC3743797

[B187] Dell’Agli M Galli GV Bulgari M Basilico N Romeo S Bhattacharya D Ellagitannins of the fruit rind of pomegranate (Punica granatum) antagonize *in vitro* the host inflammatory response mechanisms involved in the onset of malaria Malar J 2010 9 208 20642847 10.1186/1475-2875-9-208 PMC2912927

[B188] Prato M Malarial pigment does not induce MMP-2 and TIMP-2 protein release by human monocytes Asian Pac J Trop Med 2011 4 756 21967702 10.1016/S1995-7645(11)60187-9

[B189] Prato M Gallo V Arese P Higher production of tumor necrosis factor alpha in hemozoin-fed human adherent monocytes is dependent on lipidic component of malarial pigment: new evidences on cytokine regulation in P. *falciparum* malaria Asian Pac J Trop Med 2010 3 85 89

[B190] Polimeni M Valente E Aldieri E Khadjavi A Giribaldi G Prato M Role of 15-hydroxyeicosatetraenoic acid in hemozoin-induced lysozyme release from human adherent monocytes Biofactors 2013 39 304 314 23355332 10.1002/biof.1071

[B191] Polimeni M Valente E Aldieri E Khadjavi A Giribaldi G Prato M Haemozoin induces early cytokine-mediated lysozyme release from human monocytes through p38 MAPK- and NF-kappaB-dependent mechanisms PLoS One 2012 7 e39497 22724024 10.1371/journal.pone.0039497 PMC3377659

[B192] Prato M Giribaldi G Arese P Hemozoin triggers tumor necrosis factor alpha-mediated release of lysozyme by human adherent monocytes: new evidences on leukocyte degranulation in P. falciparum malaria Asian Pac J Trop Med 2009 2 3 35 40

[B193] Geurts N Martens E Van Aelst I Proost P Ulliers D Opdenakker G Van den Steen PE Beta-hematin interaction with the hemopexin domain of gelatinase B/MMP-9 provokes autocatalytic processing of the propeptide, thereby priming activation by MMP-3 Biochemistry 2008 47 2689 2699 18237197 10.1021/bi702260q

[B194] Prato M D’Alessandro S Van den Steen PE Opdenakker G Arese P Taramelli D Basilico N Natural haemozoin modulates matrix metalloproteinases and induces morphological changes in human microvascular endothelium Cell Microbiol 2011 13 1275 1285 21707906 10.1111/j.1462-5822.2011.01620.x

[B195] D’Alessandro S Basilico N Prato M Effects of Plasmodium falciparum-infected erythrocytes on matrix metalloproteinase-9 regulation in human microvascular endothelial cells Asian Pac J Trop Med 2013 6 195 199 23375032 10.1016/S1995-7645(13)60022-X

[B196] Fauser S Deininger MH Kremsner PG Magdolen V Luther T Meyermann R Lesion associated expression of urokinase-type plasminogen activator receptor (uPAR, CD87) in human cerebral malaria J Neuroimmunol 2000 111 234 240 11063844 10.1016/s0165-5728(00)00368-4

[B197] Deininger MH Winkler S Kremsner PG Meyermann R Schluesener HJ Angiogenic proteins in brains of patients who died with cerebral malaria J Neuroimmunol 2003 142 101 111 14512169 10.1016/s0165-5728(03)00250-9

[B198] Deininger MH Fimmen B Kremsner PG Meyermann R Schluesener HJ Accumulation of endostatin/collagenXVIII in brains of patients who died with cerebral malaria J Neuroimmunol 2002 131 216 221 12458056 10.1016/s0165-5728(02)00276-x

[B199] Griffiths MJ Shafi MJ Popper SJ Hemingway CA Kortok MM Wathen A Genomewide analysis of the host response to malaria in Kenyan children J Infect Dis 2005 191 1599 1611 15838786 10.1086/429297

[B200] Dietmann A Helbok R Lackner P Issifou S Lell B Matsiegui PB Matrix metalloproteinases and their tissue inhibitors (TIMPs) in Plasmodium falciparum malaria: serum levels of TIMP-1 are associated with disease severity J Infect Dis 2008 197 1614 1620 18700258 10.1086/587943

[B201] Noone C Parkinson M Dowling DJ Aldridge A Kirwan P Molloy SF Plasma cytokines, chemokines and cellular immune responses in pre-school Nigerian children infected with Plasmodium falciparum Malar J 2013 12 5 23294670 10.1186/1475-2875-12-5 PMC3545738

[B202] Jung K Measurement of matrix metalloproteinases and their tissue inhibitors in serum produces doubtful results J Infect Dis 2008 198 1722 1723 19000015 10.1086/593070

[B203] Giebel SJ Menicucci G McGuire PG Das A Matrix metalloproteinases in early diabetic retinopathy and their role in alteration of the blood-retinal barrier Lab Invest 2005 85 597 607 15711567 10.1038/labinvest.3700251

[B204] Gurney KJ Estrada EY Rosenberg GA Blood–brain barrier disruption by stromelysin-1 facilitates neutrophil infiltration in neuroinflammation Neurobiol Dis 2006 23 87 96 16624562 10.1016/j.nbd.2006.02.006

[B205] Gorodeski GI Estrogen decrease in tight junctional resistance involves matrix-metalloproteinase-7-mediated remodeling of occludin Endocrinology 2007 148 218 231 17038551 10.1210/en.2006-1120 PMC2398688

[B206] Chiu PS Lai SC Matrix metalloproteinase-9 leads to claudin-5 degradation via the NF-κB pathway in BALB/c mice with eosinophilic meningoencephalitis caused by Angiostrongylus cantonensis PLoS One 2013 8 e53370 23505411 10.1371/journal.pone.0053370 PMC3591436

[B207] Liu J Jin X Liu KJ Liu W Matrix metalloproteinase-2-mediated occludin degradation and caveolin-1-mediated claudin-5 redistribution contribute to blood–brain barrier damage in early ischemic stroke stage J Neurosci 2012 32 3044 3057 22378877 10.1523/JNEUROSCI.6409-11.2012 PMC3339570

[B208] Feng S Cen J Huang Y Shen H Yao L Wang Y Chen Z Matrix metalloproteinase-2 and -9 secreted by leukemic cells increase the permeabilità of blood-brain barrier by disrupting tight junction proteins PLoS One 2011 6 e20599 Erratum in: *PLoS One* 2011; 6(8) 21857898 10.1371/journal.pone.0020599 PMC3157343

[B209] Qiu LB Zhou Y Wang Q Yang LL Liu HQ Xu SL Qi YH Ding GR Guo GZ Synthetic gelatinases inhibitor attenuates electromagnetic pulse-induced blood–brain barrier disruption by inhibiting gelatinases-mediated ZO-1 degradation in rats Toxicology 2011 285 31 38 21501651 10.1016/j.tox.2011.03.019

[B210] Lijnen HR Elements of the fibrinolytic system Ann N Y Acad Sci 2001 936 226 236 11460480 10.1111/j.1749-6632.2001.tb03511.x

[B211] Lijnen HR Matrix metalloproteinases and cellular fibrinolytic activity Biochemistry 2002 67 92 98 11841344 10.1023/a:1013908332232

[B212] Santos-Martínez MJ Medina C Jurasz P Radomski MW Role of metalloproteinases in platelet function Thromb Res 2008 121 535 542 17681591 10.1016/j.thromres.2007.06.002

[B213] Lesiak A Narbutt J Sysa-Jedrzejowska A Lukamowicz J McCauliffe DP Wózniacka A Effect of chloroquine phosphate treatment on serum MMP-9 and TIMP-1 levels in patients with systemic lupus erythematosus Lupus 2010 19 683 688 20064914 10.1177/0961203309356455

[B214] Buommino E Baroni A Canozo N Petrazzuolo M Nicoletti R Vozza A Tufano MA Artemisinin reduces human melanoma cell migration by down-regulating alpha V beta 3 integrin and reducing metalloproteinase 2 production Invest New Drugs 2009 27 412 418 18956140 10.1007/s10637-008-9188-2

[B215] Wartenberg M Wolf S Budde P Grünheck F Acker H Hescheler J Wartenberg G Sauer H The antimalaria agent artemisinin exerts antiangiogenic effects in mouse embryonic stem cell-derived embryoid bodies Lab Invest 2003 83 1647 1655 14615418 10.1097/01.lab.0000098424.38003.ff

[B216] Hwang YP Yun HJ Kim HG Han EH Lee GW Jeong HG Suppression of PMA-induced tumor cell invasion by dihydroartemisinin via inhibition of PKCalpha/Raf/MAPKs and NF-kappaB/AP-1-dependent mechanisms Biochem Pharmacol 2010 79 1714 1726 20152819 10.1016/j.bcp.2010.02.003

[B217] Wang SJ Sun B Cheng ZX Zhou HX Gao Y Kong R Chen H Jiang HC Pan SH Xue DB Bai XW Dihydroartemisinin inhibits angiogenesis in pancreatic cancer by targeting the NF-κB pathway Cancer Chemother Pharmacol 2011 68 1421 1430 21479633 10.1007/s00280-011-1643-7

[B218] Rasheed SA Efferth T Asangani IA Allgayer H First evidence that the antimalarial drug artesunate inhibits invasion and *in vivo* metastasis in lung cancer by targeting essential extracellular proteases Int J Cancer 2010 127 1475 1485 20232396 10.1002/ijc.25315

[B219] Peterson JT The importance of estimating the therapeutic index in the development of matrix metalloproteinase inhibitors Cardiovasc Res 2006 69 677 687 16413004 10.1016/j.cardiores.2005.11.032

[B220] Konstantinopoulos PA Karamouzis MV Papatsoris AG Papavassiliou AG Matrix metalloproteinase inhibitors as anticancer agents Int J Biochem Cell Biol 2008 40 1156 1168 18164645 10.1016/j.biocel.2007.11.007

[B221] Dormán G Cseh S Hajdú I Barna L Kónya D Kupai K Matrix metalloproteinase inhibitors: a critical appraisal of design principles and proposed therapeutic utility Drugs 2010 70 949 964 20481653 10.2165/11318390-000000000-00000

[B222] Murphy G Nagase H Progress in matrix metalloproteinase research Mol Aspects Med 2008 29 290 308 18619669 10.1016/j.mam.2008.05.002 PMC2810947

[B223] Hu J Van den Steen PE Sang QX Opdenakker G Matrix metalloproteinase inhibitors as therapy for inflammatory and vascular diseases Nat Rev Drug Discov 2007 6 480 498 17541420 10.1038/nrd2308

[B224] Pirard B Insight into the structural determinants for selective inhibition of matrix metalloproteinases Drug Discov Today 2007 12 640 646 17706545 10.1016/j.drudis.2007.06.003

[B225] Devel L Czarny B Beau F Georgiadis D Stura E Dive V Third generation of matrix metalloprotease inhibitors: gain in selectivity by targeting the depth of the S1′ cavity Biochimie 2010 92 1501 1508 20696203 10.1016/j.biochi.2010.07.017

[B226] Fingleton B MMPs as therapeutic targets–still a viable option? Semin Cell Dev Biol 2008 19 61 68 17693104 10.1016/j.semcdb.2007.06.006 PMC2677300

[B227] Murata CE Goldberg DE Plasmodium falciparum falcilysin: a metalloprotease with dual specificity J Biol Chem 2003 278 38022 38028 12876284 10.1074/jbc.M306842200

